# Assessment of the Optimum Linker Tethering Site of Alternariol Haptens for Antibody Generation and Immunoassay Development

**DOI:** 10.3390/toxins13120883

**Published:** 2021-12-10

**Authors:** Luis G. Addante-Moya, Antonio Abad-Somovilla, Antonio Abad-Fuentes, Consuelo Agulló, Josep V. Mercader

**Affiliations:** 1 Department of Organic Chemistry, University of Valencia, Doctor Moliner 50, 46100 Burjassot, Valencia, Spain; luis.addante@uv.es (L.G.A.-M.); antonio.abad@uv.es (A.A.-S.); consuelo.agullo@uv.es (C.A.); 2 Spanish Council for Scientific Research, Institute of Agrochemistry and Food Technology, Agustí Escardino 7, 46980 Paterna, Valencia, Spain; aabad@iata.csic.es

**Keywords:** alternariol, antibody, ELISA, hapten design, immunoassay, linker site

## Abstract

Immunochemical methods for mycotoxin analysis require antigens with well-defined structures and antibodies with outstanding binding properties. Immunoreagents for the mycotoxins alternariol and/or alternariol monomethyl ether have typically been obtained with chemically uncharacterized haptens, and antigen conjugates have most likely been prepared with mixtures of functionalized molecules. For the first time, total synthesis was performed, in the present study, to obtain two haptens with opposite linker attachment locations. The functionalized synthetic haptens were purified and deeply characterized by different spectrometric methods, allowing the preparation of bioconjugates with unequivocal structures. Direct and indirect competitive enzyme-linked immunosorbent assays, using homologous and heterologous conjugates, were employed to extensively evaluate the generated immunoreagents. Antibodies with high affinity were raised from conjugates of both haptens, and a structure-activity relationship between the synthetic haptens and the specificity of the generated antibodies could be established. These results pave the way for the development of novel highly sensitive immunoassays selective of one or two of these *Alternaria* mycotoxins.

## 1. Introduction

*Alternaria* sp. fungi, particularly *A. alternata*, are ubiquitous plant pathogens and saprophytes that infect economically relevant crops such as cereals, vegetables, oilseeds, and fruits. Moreover, these microorganisms can contaminate these commodities after harvest even under refrigeration conditions. They are known to produce a wide variety of toxic secondary metabolites [1], and some of them have been identified by the EFSA Panel on Contaminants in the Food Chain (CONTAM) as a potential risk to human and animal health due to their toxicity and occurrence in food and feed. Surprisingly, there are no specific international regulations for any of the *Alternaria* mycotoxins, and the available data on toxicity, occurrence, and dietary exposure are still limited. In 2011, EFSA carried out the first assessment of the risk of these mycotoxins to human and animal health, based on government and published data [2]. More recently, EFSA conducted a survey on the dietary exposure of European consumers to *Alternaria* toxins [3]. This study found that 8% of these mycotoxins are present in food, with infants and other children being the most exposed population group, and fruit and fruit-based products contributing most to dietary exposure. It is therefore expected that the European Commission will soon set maximum levels for the most common *Alternaria* mycotoxins in foodstuffs.

Alternariol (AOH) and alternariol monomethyl ether (AME), two of the most important compounds belonging to the group of *Alternaria* mycotoxins, appear to be responsible for the teratogenic effects observed in animals. They have also been shown to inhibit in vitro the catalytic topoisomerase activity, which may be associated with human colon and oesophageal cancer [4]. Frequently, these two mycotoxins are found together in samples because they share most of the biosynthetic pathway [5]. AOH and AME have been detected in a wide variety of products, including lentils, carrots, tomatoes, berries, apples, pears, beer, wines, juices, and various grains and flours [6]. To evaluate the relative hazard level of these toxins to human health, a threshold of toxicological concern (TTC) for AOH and AME in adults of 2.5 ng/kg body weight per day was established as a reference parameter by the CONTAM Panel [2]. With limited data available, a 2016 German survey concluded that the percentage of TTC reached by the average adult daily exposure was 1400% and 280% for AOH and AME, respectively [7].

A variety of analytical techniques have been developed for monitoring *Alternaria* toxins in food, including liquid and gas chromatographic methods coupled to several detection systems, as well as different types of immunochemical assays [8,9]. Molecular affinity techniques nowadays represent alternative strategies for rapid, economical, and/or on-site monitoring of mycotoxins. The first antibodies and immunoassays for AOH were reported in 2011 [10,11]. Since then, a few immunoassays have been described using either polyclonal or monoclonal antibodies specific to AOH [12,13], and only one study has been published using an antibody specific to AME [14]. Additionally, Wang et al. have reported a generic antibody for both mycotoxins [15]. In all these studies, the immunogens used to generate antibodies against AOH were made by attaching the mycotoxin to the carrier protein, either directly by a Mannich-type reaction or by carbodiimide-mediated chemistry after nonselective carboxymethylation of the hydroxyl groups. Neither of these methods can be used to guide the position of attachment of the AOH molecular scaffold to the carrier proteins, so the antibodies were actually generated from an undefined mixture of functionalized haptens. More recently, Yao et al. published an immunoassay for AOH using a carboxymethyl hapten to generate polyclonal antibodies [16]. Disappointingly, no synthetic details and spectrometric data of the prepared hapten were provided in that work.

AOH and AME are the two most representative mycotoxins with a tricyclic benzochromenone chemical backbone. Moreover, these compounds contain hydroxyl, methyl, and other substituents in their chemical structure. It is well known that the orientation of the molecule, i.e., the way the hapten is displayed to the immune system, strongly influences the specificity of the generated antibodies. Thus, the synthesis of haptens with the optimal linker tethering site is critical, although it is hard to predict and challenging to perform. Surprisingly, deeply characterized haptens for AOH or AME with unambiguous chemical structures have not been published so far. The aim of the present study was to prepare, purify, and characterize two rationally-designed synthetic haptens of these *Alternaria* mycotoxins with a functionalized linker located at precise sites of the molecule. The ability of these novel immunoreagents to elicit a potent immune response, ultimately leading to high-affinity antibodies, was investigated. In addition, these bioconjugates also allowed the study of the relationship between the functionalization position in the hapten and the specificity of the resulting antibodies.

## 2. Results and Discussion

### 2.1. Hapten Design and Synthesis

**Figure 1:**
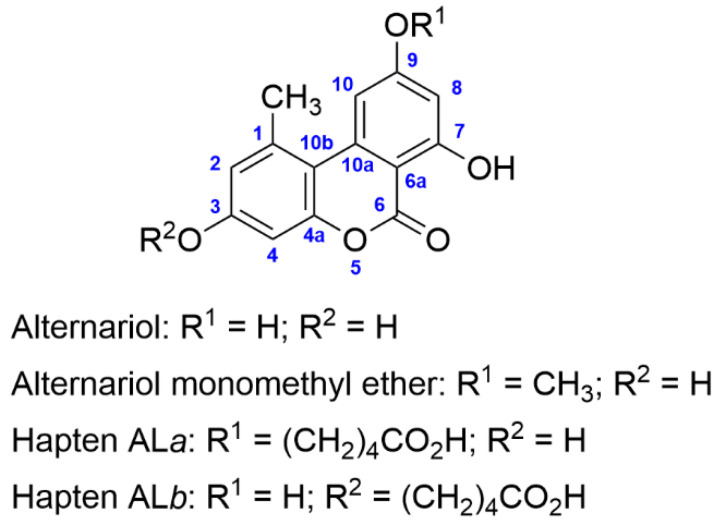
Chemical structures of alternariol, alternariol monomethyl ether, and the synthetic haptens.

The generation of antibodies to AOH has so far been based on the preparation of the required immunogens from AOH itself, which does not allow fine control over the specific position of the mycotoxin framework where the functionalized linker is introduced. In this study, we have synthesized two regioisomeric haptens of AOH from scratch. One of them, hapten **AL*a***, incorporates a five-atom carboxylated aliphatic spacer arm through the hydroxyl group at C-9, whereas the other one, hapten **AL*b***, incorporates the same linker via the hydroxyl group at C-3 (Figure 1). In contrast to previous strategies, these two haptens allowed the preparation of bioconjugates with well-defined compositions.

**Scheme 1:**
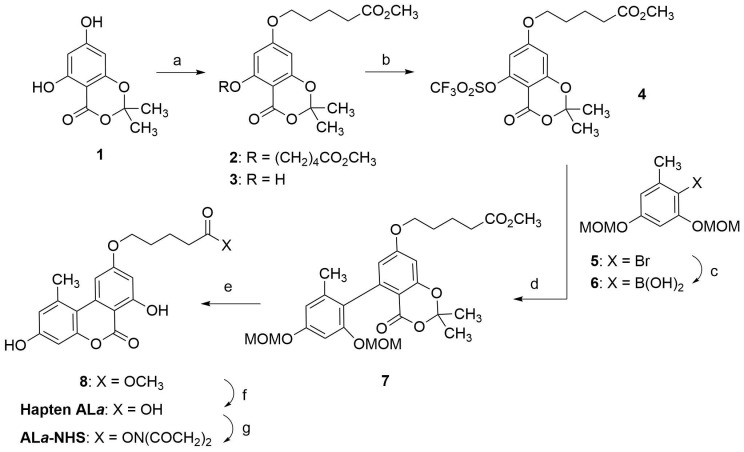
Synthesis of **AL*a*-NHS** ester. Reagents and conditions: (a) Br(CH_2_)_4_CO_2_CH_3_, K_2_CO_3_, KI, Bu_4_NBr, acetone, reflux, 16 h, 75% of **3**. (b) Tf_2_O, pyridine, 0 °C to rt, 20 h, 91%. (c) i. n-BuLi, THF, −78 °C, 40 min; ii. B(O^i^Pr)_3_, −78 °C to 0 °C, 1.5 h, 93%. (d) Pd(PPh_3_)_4_, K_2_CO_3_, DMF, 93 °C, 24 h, 75%. (e) i. HCl, MeOH, rt, 22 h; ii. TFA, CH_2_Cl_2_, rt, 20 h, 97%. (f) Lipase acrylic resin, THF-PB 100 mM, rt, 20 h, 93%. (g) EDC·HCl, NHS, DMF, rt, overnight, 99% of crude product.

The synthetic strategy for preparing hapten **AL*a*** was based on a convergent methodology previously used by several research groups to synthesize AOH and other structurally related molecules [17,18,19,20]. A key step in this synthesis is a Pd(0)-catalyzed cross-coupling reaction between an aryl triflate (**4**), which already contained the spacer arm, and an appropriately functionalized arylboronic acid (**6**) (Scheme 1). The aryl triflate **4** was prepared in two steps from the readily available 1,3-benzodioxinone **1** [21,22]. First, an *O*-alkylation reaction with methyl 5-bromovalerate was performed under standard Williamson ether synthesis conditions. The alkylation process produced a 6:1 mixture of di- and mono-*O*-alkylation products, **2** and **3**, respectively, which were easily separated by column chromatography to provide the product resulting from the selective *O*-alkylation of the less hindered hydroxyl group, i.e., **3**, with a 75% yield. The free hydroxyl group of **3** was then converted to the required triflate group by reaction with triflic anhydride in pyridine, giving the triflate **4** in 91% yield. The additional required coupling reactant, aryl boronic acid **6**, was prepared from the orcinol-derived bromide **5** [23] by halogen-metal exchange using butyllithium and reaction of the resulting lithiated derivative with triisopropyl borate.

The subsequent palladium-catalyzed Suzuki-Miyaura coupling between the aryl triflate **4** and the labile boronic acid **6** gave the biaryl **7** in 75% yield. Hydrolysis of the methoxymethyl ether (MOM) groups by treatment with methanolic HCl, followed by intramolecular transesterification promoted by trifluoroacetic acid, completed the synthesis of the tricyclic benzochromenone backbone and afforded the methyl ester of hapten **AL*a***, compound **8**, in 97% yield. To complete the synthesis of hapten **AL*a***, only the hydrolysis of the methyl ester moiety of **8** was required, which was initially carried out under basic conditions (LiOH in THF-H_2_O at room temperature, rt). However, under these conditions, the central lactone group of the benzochromenone core was partially opened, requiring acid treatment of the reaction crude to reconstruct the tricyclic ring system. It proved more convenient to carry out this transformation using enzymatic hydrolysis, so a lipase from *Candida antarctica* immobilized on an acrylic resin was used to hydrolyze the methyl ester group, providing hapten **AL*a*** in practically quantitative yield.

Upon completion of the synthesis of hapten **AL*a***, its carboxylic group was activated by forming the corresponding *N*-hydroxysuccinimidyl ester. This transformation was carried out under conventional activation conditions, with *N*-(3-dimethylaminopropyl)-*N*′-ethylcarbodiimide hydrochloride (EDC·HCl) and *N*-hydroxisuccinimide (NHS) in *N*,*N*-dimethylformamide (DMF) at rt, yielding the corresponding *N*-hydroxysuccinimidyl ester, **AL*a*-NHS**, in good yield. The activated hapten was extracted essentially pure from the reaction as judged by ^1^H NMR, so it was further used without additional purification by column chromatography. NMR spectra of all of the intermediates and the hapten can be found in the Supplementary Materials file.

**Scheme 2:**
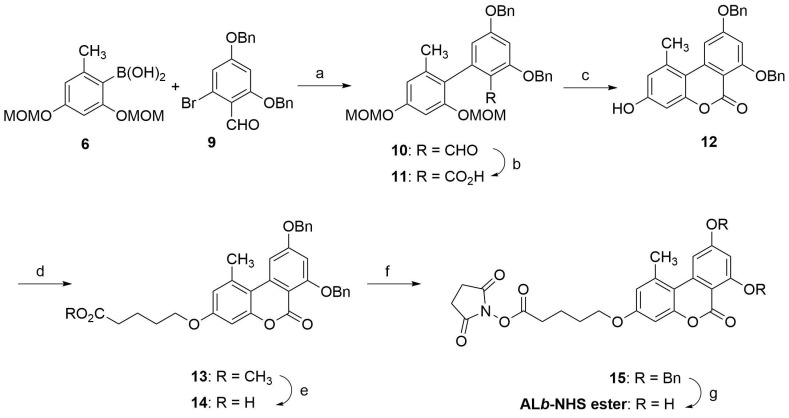
Synthesis of **AL*b*-NHS** ester. Reagents and conditions: (a) Pd(PPh_3_)_4_, K_2_CO_3_, DMF, 95 °C, 19 h, 77%. (b) NaH_2_PO_4_·H_2_O, NaClO_2_, ^t^BuOH-H_2_O (5:1), rt, 5 h, 96%. (c) ^i^PrOH, THF, conc HCl, 55 °C, 24 h, 98%. (d) Br(CH_2_)_4_CO_2_CH_3_, Cs_2_CO_3_, DMF, 94%. (e) Lipase acrylic resin, THF-PB 100 mM, rt, 20 h, 99%. (f) EDC·HCl, NHS, DMF, rt, overnight. (g) 5% Pd/C, acetone, H_2_ (1.5 atm), rt, 19 h, 95% of crude product from **14**.

Hapten **AL*b*** was synthesized following a similar procedure as hapten **AL*a***, except that in this case the tricyclic benzochromenone core was built first, with the hydroxyl groups appropriately protected to allow subsequent incorporation of the spacer arm at the required C-3 position. As shown in Scheme 2, the synthesis of the benzochromenone ring system began with the palladium-catalyzed cross-coupling reaction between the aryl boronic acid **6** and the previously reported bromobenzaldehyde **9** [24,25]. This coupling was carried out under conditions similar to those previously used for the conversion of **4** and **6** into **7**, obtaining the biphenyl-2-carbaldehyde **10** in 77% yield. The formyl group was further oxidized to the carboxylic group under Pinnick oxidation conditions to afford the biphenyl-2-carboxylic acid **11**, which was then treated with methanolic HCl at 55 °C to promote deprotection of the MOM groups and further intramolecular esterification, thus completing the formation of the tricyclic benzochromenone system. Under these conditions, both sequential processes worked extremely well, affording **12** in practically quantitative yield. *O*-alkylation of the phenol-like hydroxyl group at the C-3 position of **12** with methyl 5-bromovalerate, using Cs_2_CO_3_ in DMF as base, gave the *O*-alkylated derivative **13** in 94% yield. The methyl ester of **13** was further converted to the corresponding carboxylic group under enzymatic hydrolytic conditions, yielding **14** also in high yield. The hapten **AL*b*** was first obtained by hydrogenolysis of both benzyl ether groups of **14** using 5% Pd on activated carbon as catalyst. With hapten **AL*b*** in hand, we activated the carboxylic group using the carbodiimide-NHS procedure as was done for hapten **AL*a***. However, the overall yield from these two processes was low, most likely motivated by an intermolecular esterification reaction between a hydroxyl group and the aliphatic active ester that resulted in the spontaneous formation of a transparent thin film, a polyester polymer, on the flask walls. By reversing the order of these steps, i.e., by activating the carboxylic group first and then releasing the hydroxyl groups, a much better result was obtained. Thus, treatment of carboxylic acid **14** with EDC and NHS as before, followed by hydrogenolysis of the benzyl ether moieties of the resulting *N*-hydroxysuccinimidyl ester **15** with 5% Pd on activated carbon in acetone, gave the desired *N*-hydroxysuccinimidyl ester of hapten **AL*b***, **AL*b*-NHS** ester, with a very high overall yield. As in the case of the active ester of hapten **AL*a***, the **AL*b*-NHS** ester was extracted essentially pure from the reaction as judged by ^1^H NMR, so it was further used without additional purification by column chromatography. NMR spectra of all of the intermediates and the hapten can be found in the Supplementary Materials file.

### 2.2. Bioconjugate Preparation

**Figure 2:**
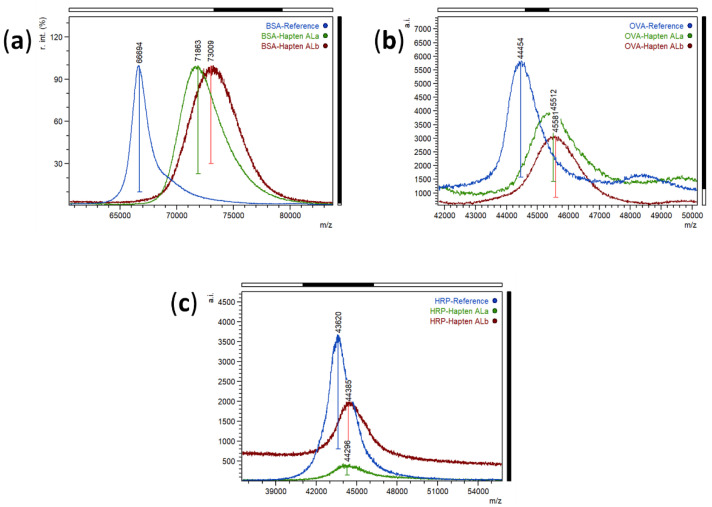
MALDI-TOF mass spectra (singly charged ions) of proteins (blue) and bioconjugates with hapten **AL*a*** (green) and hapten **AL*b*** (brick-red). (**a**) Normalized spectra of BSA and BSA conjugates, (**b**) Spectra of OVA and OVA conjugates, and (**c**) Spectra of HRP and HRP conjugates.

Bioconjugates of haptens **AL*a*** and **AL*b*** were prepared by the active ester method. The activated haptens were dissolved in dimethyl sulfoxide (DMSO) instead of DMF to improve the solubility. Moreover, the number of hapten equivalents required for efficiently labelling the studied proteins was higher than usual. Commonly, 20-fold hapten-to-protein molar excess for bovine serum albumin (BSA), and 10-fold excess for ovalbumin (OVA) and horseradish peroxidase (HRP) are usually employed in our laboratory. For these haptens, 40-fold and 15-fold excess was used for BSA and HRP conjugates, respectively. Moreover, extremely slow addition of the hapten over the protein solution was required. These concentrations and procedures were necessary, probably due to low hapten solubility in buffer and potential intermolecular polymerization reactions that inactivate the hapten. The obtained bioconjugates were purified by size-exclusion chromatography and characterized by matrix-assisted laser desorption ionization time-of-flight mass spectrometry (MALDI-TOF/MS) analysis. The two BSA conjugates had similar hapten densities, with hapten-to-protein molar ratios of 15.2 and 18.6 for BSA-**AL*a*** and BSA-**AL*b***, respectively, which is considered optimal for immunogens—excessive molar ratios could lead to low protein solubility, and higher or lower hapten densities could be counter-productive for high-affinity antibody generation. Regarding ovalbumin (OVA) conjugates, molar ratios were lower than those of BSA conjugates—around 3 for both haptens –, as it is desirable for coating conjugates to enhance the competitive reaction with the target analyte. Finally, the hapten densities of the enzyme tracers were estimated to be 2.0 and 2.2 for haptens **AL*a*** and **AL*b***, respectively, which is within the expected range for HRP conjugates. The MALDI spectra of the prepared bioconjugates can be seen in Figure 2.

### 2.3. Assessment of the Immune Response

Four polyclonal antibodies were generated in this study, two from each BSA-hapten conjugate. To evaluate the immune response to the prepared synthetic haptens, binding of the antibodies to the homologous conjugate—the conjugate with the same hapten that was used to generate the corresponding antibody—was studied by checkerboard competitive ELISA, using the direct and the indirect assay formats.

Concerning direct assays, the IC_50_ values for AOH of the obtained antibodies were in the low nanomolar range (Table 1). AL*a*-type antibodies showed equal or similar IC_50_ values for AOH and AME. In particular, antibody AL*a*#1 showed very high affinity—IC_50_ values were 2.2 nM—for both mycotoxins, and the cross-reactivity (CR) values of antibodies AL*a*#1 and AL*a*#2 for AME were 100% and 199%, respectively. These are the first reported polyclonal antibodies with equivalent recognition to both *Alternaria* toxins. To date, only one monoclonal antibody with such specificity has been published [15]. In contrast, AL*b*-type antibodies bound AOH with high affinity, but their recognition for AME was negligible—CR values were below 1% (Table 1). The IC_50_ values to AOH of these specific antibodies were 1.2 nM, an affinity comparable to that of previously published polyclonal antibodies [11,13,16]. The position of the spacer arm in hapten **AL*a*** provided a closer mimic of the alkylated hydroxyl group of AME (C-9 position), whereas in hapten **AL*b*** the hydroxyl groups at C-7 and C-9 were unsubstituted, as in the molecule of AOH (Figure 1). Therefore, display of the hydroxyl group at C-9 was maximized in hapten **AL*b***, which explains the much lower affinity of AL*b*-type antibodies for AME compared to AOH.

Regarding the indirect assay format, the four antibodies bound the corresponding homologous coating conjugate. As observed with the direct format, the AL*a*-derived antibodies recognized AOH and AME, whereas the AL*b*-derived antibodies were more specific to AOH (Table 1). The IC_50_ values were consistent with previously published results for indirect competitive ELISA with polyclonal antibodies [10,11,16]. Our strategy to prepare immunizing haptens with opposite linker tethering sites clearly demonstrated that the linker position strongly determines the specificity of antibodies to these *Alternaria* mycotoxins.

### 2.4. Assessment of Heterologous Conjugates

Heterologous conjugates constitute a well-known strategy for improving the sensitivity of immunoassays. To further characterize the generated antibodies, competitive assays were carried out using the heterologous conjugate, i.e., assay conjugates of haptens **AL*a*** and **AL*b*** for AL*b*- and AL*a*-type antibodies, respectively. In the direct assay format, low binding to the heterologous tracer—with the linker on the opposite side of the AOH molecule compared to the immunizing conjugate—was observed (A_max_ values were below 0.6). In contrast, the change in the linker attachment site was not detrimental to hapten recognition in the indirect format, as the four antibodies bound the corresponding heterologous coating conjugate (Table 2). Reasonably, higher antibody and/or conjugate concentrations were required with the heterologous conjugates to reach sufficient signal. The obtained IC_50_ values using the heterologous coating conjugate were mostly lower than those obtained with the homologous assays. Anyway, CR values did not significantly change with heterologous conjugates.

## 3. Conclusions

In this study, two de novo synthesized and purified AOH haptens were comprehensively characterized by spectrometric methods, and bioconjugates with unique structure and composition were prepared for the first time. In this perspective, it is worth noting the challenges of obtaining stable enzyme tracers with high activity. This matter was most likely caused by the chemical characteristics of *Alternaria* toxins and their haptens, which could explain why no direct competitive immunoassays for these mycotoxins have been reported up to now. Once this issue was overcome, the resultant immunoreagents were thoroughly investigated utilizing both direct and indirect competitive ELISA, as well as homologous and heterologous conjugates. Remarkably, antibodies capable of binding AOH and AME with affinities in the low nanomolar range were eventually generated from both haptens. Given that the levels of these mycotoxins are not yet regulated, both specific and generic antibodies are relevant. Our findings showed that hapten **AL*a***, with the linker at the methylated hydroxyl group in AME (C-9 position), was particularly well-suited for producing antibodies that recognized similarly both toxins, whereas antibodies generated from hapten **AL*b***, with the spacer arm at the hydroxyl group in C-3 position, primarily bound AOH. In contrast to previous one-pot hapten synthesis and bioconjugation procedures, the strategy described here for producing AOH haptens with alternative linker tethering sites not only enabled high-affinity antibodies with different specificities, but it may also help to improve the sensitivity of immunoassays to *Alternaria* mycotoxins by using site heterologous haptens.

## 4. Materials and Methods

### 4.1. Reagents and Instruments

Standard AOH [3,7,9-trihydroxy-1-methyl-benzo[c]chromen-6-one, CAS registry number 641-38-3, Mw 258.23] and AME [3,7-dihydroxy-9-methoxy-1-methyl-benzo[c]chromen-6-one, CAS registry number 23452-05-3, Mw 272.25] from *Alternaria* sp. were purchased from Merck (Darmstadt, Germany). Mycotoxins were dissolved in anhydrous *N*,*N*-dimethylformamide (DMF), and the stock solutions were stored at −20 °C. Phosphate buffered saline (PBS) 10× solution (Fisher BioReagents BP399-20) was from Thermo Fisher Scientific (Waltham, MA, USA). Immunizing bioconjugates were prepared with BSA, fraction V, obtained from Roche Applied Science (Mannheim, Germany). OVA, HRP, Freund’s adjuvants, and adult bovine serum, were acquired from Merck (Darmstadt, Germany). Polyclonal goat anti-rabbit (GAR) immunoglobulins antibody and polyclonal goat anti-rabbit immunoglobulins antibody conjugated to peroxidase (GAR-HRP) were purchased from Rockland Immunochemicals Inc. (Pottstown, PA, USA) and BioRad (Madrid, Spain), respectively. 3,3′,5,5′-Tetramethylbenzidine (TMB) liquid substrate for ELISA was obtained from Biopanda Reagents Ltd. (Belfast, UK). Other reagents, materials, and instruments employed for bioconjugate preparation and ELISA experiments are described in the Supplementary Materials file.

### 4.2. Synthesis of the N-hydroxysuccinimidyl Ester of Hapten **ALa**

#### 4.2.1. Preparation of methyl 5-((5-hydroxy-2,2-dimethyl-4-oxo-4H-benzo[d][1,3]dioxin-7-yl)oxy)pentanoate (**3**)

Methyl 5-bromovalerate (186 µL, 266 mg, 1.36 mmol, 1.1 equiv) was added to a solution of 1,3-benzodioxinone **1** (260 mg, 1.237 mmol), KI (83 mg, 0.500 mmol, 0.4 equiv), Bu_4_NBr (0.5 mg, 1.6 µmol) y K_2_CO_3_ (188 mg, 1.36 mmol, 1 equiv) in dry acetone (9 mL) under nitrogen. After heating the mixture at reflux for 16 h, the acetone was eliminated at reduced pressure and the resulting brownish residue was diluted with water and extracted with Et_2_O. The combined organic layers were washed with water and brine, dried over anhydrous MgSO_4_ and concentrated under vacuum. The obtained crude product was purified by chromatography on silica gel, using hexane-EtOAc mixtures from 9:1 to 7:3 as eluent, to afford, in order of elution, dialkylated derivative **2** (67.7 mg, 12.5%) and monoalkylated compound **3** (300 mg, 75%) as a white solid. Mp 97.3–98.2 °C (crystallized from hexane-EtOAc) IR (ATR) ν_max_ (cm^−1^) 3017 (w), 1740 (s), 1672 (s), 1251 (s), 1159 (s), 840 (s), 794 (s); ^1^H NMR (300 MHz, CDCl_3_) δ 10.42 (s, 1H, OH), 6.11 (d, *J* = 2.2 Hz, 1H, H-6), 5.97 (d, *J* = 2.2 Hz, 1H, H-8), 3.98 (t, *J* = 5.8 Hz, 2H, H_2_-5), 3.68 (s, 3H, OCH_3_), 2.39 (t, *J* = 7.0 Hz, 2H, H_2_-2), 1.81 (m, 4H, H_2_-3 and H_2_-4), 1.72 (s, 6H, 2×CH_3_); ^13^C NMR (75 MHz, CDCl_3_) δ 173.8 (CO_2_CH_3_), 167.2 (CO), 165.3 (C-7), 163.2 (C-8a), 156.9 (C-5), 107.0 (C-2), 96.3 (CH-6), 95.1 (CH-8), 93.1 (C-4a), 68.1 (CH_2_-5), 51.7 (OCH_3_), 33.7 (CH_2_-2), 28.4 (CH_2_-4), 25.8 (2×CH_3_), 21.6 (CH_2_-3); HRMS (TOF MS ES+) *m/z* calculated for C_16_H_20_O_7_ [M + H]^+^ 325.1282, found 325.1283.

#### 4.2.2. Preparation of methyl 5-((2,2-dimethyl-4-oxo-5-(((trifluoromethyl)sulfonyl)oxy)-4H-benzo[d][1,3]dioxin-7-yl)oxy)pentanoate (**4**)

Triflic anhydride (230 µL, 1.369 mmol, 1.5 equiv) was added to a solution of phenol **3** (296 mg, 0.913 mmol) in anhydrous pyridine (4.5 mL) at 0 °C under nitrogen. The reaction mixture was allowed to warm to rt and stirred for 20 h, then cooled down to 0 °C and treated with a saturated aqueous solution of NaHCO_3_, stirred for a few minutes at rt and then extracted with Et_2_O. The organic layers were washed with water, a 1% (*w*/*v*) aqueous solution of CuSO_4_ and brine, dried over anhydrous MgSO_4_ and concentrated at reduced pressure. The obtained residue was chromatographed on silica gel, using hexane-EtOAc mixtures from 9:1 to 8:2 as eluent, to give aryl triflate **4** (378.8 mg, 91%) as a white semisolid. IR (ATR) ν_max_ (cm^−1^) 3114 (w), 1746 (s), 1733 (s), 1381 (s), 1228 (s), 1167 (s), 869 (s); ^1^H NMR (300 MHz, CDCl_3_) δ 6.51 (d, *J =* 2.3 Hz, 1H, H-6), 6.45 (d, *J =* 2.3 Hz, 1H, H-8), 4.02 (t, *J =* 5.8 Hz, 2H, H_2_-5), 3.68 (s, 3H, CO_2_CH_3_), 2.40 (t, *J =* 6.9 Hz, 2H, H_2_-2), 1.85 (m, 4H, H_2_-3 and H_2_-4), 1.73 (s, 6H, 2xCH_3_); ^13^C NMR (75 MHz, CDCl_3_) δ 173.7 (CO_2_CH_3_), 165.0 (CO), 158.9 (C-7), 157.2 (C-8a), 150.1 (C-5), 106.7 (C-2), 105.7 (CH-6), 101.6 (CH-8), 101.0 (C-4a), 68.9 (CH_2_-5), 51.8 (OCH_3_), 33.6 (CH_2_-2), 28.3 (CH_2_-4), 25.7 (2×CH_3_), 21.5 (CH_2_-3); ^19^F NMR (282 MHz, CDCl_3_) δ 73.1 (s, CF*_3_*); HRMS (TOF, ES+) *m/z* calculated for C_17_H_23_F_3_NO_9_S [M + NH_4_]^+^ 474.1040; found 474.1027.

#### 4.2.3. Preparation of methyl 5-((5-(2,4-bis(methoxymethoxy)-6-methylphenyl)-2,2-dimethyl-4-oxo-4H-benzo[d][1,3]dioxin-7-yl)oxy)pentanoate (**7**)

(i) Preparation of boronic acid **6**. A solution of *n*-BuLi in hexane (1.3 M, 336 µL, 0.436 mmol, 1.05 equiv) was dropwise added to a solution of aryl bromide **5** (122.3 mg, 0.420 mmol) in anhydrous THF (2.5 mL) at −78 °C under nitrogen. The reaction mixture was stirred at this temperature for 40 min, B(O^i^Pr)_3_ (322 µL, 1.386 mmol, 3.3 equiv) was then added and the mixture stirred for 1.5 h. After this time, the dry ice bath was replaced by an ice bath and the mixture treated with an aqueous saturated solution of NH_4_Cl (0.7 mL), then diluted with water and extracted with Et_2_O. The organic layers were washed with brine, dried over anhydrous Na_2_SO_4_ and concentrated under reduced pressure to give boronic acid **6** (100.0 mg, 93%) as a thick oil that was immediately used in the next reaction without further purification since it is relatively prone to protodeboronation [26]. ^1^H NMR (300 MHz, DMSO-*d*_6_) δ 7.91 (s, 1H, BOH), 6.49 (d, *J =* 2.1 Hz, 1H, H-4), 6.47 (d, *J =* 2.10 Hz, 1H, H-6), 5.12 and 5.09 (each s, 2H each, 2×OCH_2_O), 3.37 and 3.34 (each s, 3H each, 2×OCH_3_), 2.20 (s, 3H, CH_3_).

(ii) Coupling reaction between aryl triflate **4** and boronic acid **6**. A mixture of the above obtained boronic acid **6** (47.4 mg, 0.185 mmol), aryl triflate **4** (41.6 mg, 0.091 mmol), powdered K_2_CO_3_ (43.2 mg, 0.312 mmol) and Pd(PPh_3_)_4_ (11.4 mg, 9.9 µmol) under nitrogen was dissolved in anhydrous DMF (1.2 mL), previously degassed by three freeze-vacuum-thaw cycles. The mixture was heated at 93 °C and stirred at this temperature for 24 h. The mixture was cooled to rt, quenched with water and extracted with EtOAc. The combined organic layers were successively washed with water, a 1.5% (*w*/*v*) aqueous solution of LiCl and brine, and dried over anhydrous MgSO_4_. The obtained residue after evaporation of the solvent was chromatographed on silica gel, using hexane-EtOAc 8:2 as eluent, to afford biaryl compound **7** (35.2 mg, 75%) as a yellowish oil. ^1^H NMR (300 MHz, CDCl_3_) δ 6.71 (d, *J* = 2.3 Hz, 1H, H-6), 6.64 (d, *J* = 2.3 Hz, 1H, H-8), 6.42 (d, *J* = 2.5 Hz, 1H, H-5), 6.40 (d, *J* = 2.5 Hz, 1H, H-3), 5.18 (AB system, *J* = 6.7 Hz, 2H, OCH_2_O), 4.98 (AB system, *J* = 6.6 Hz 2H, OCH_2_O), 3.99 (t, *J* = 5.6 Hz, 2H, H_2_-5), 3.67 (s, 3H, CO_2_CH_3_), 3.50 and 3.29 (each s, 3H each, 2×OCH_3_), 2.39 (t, *J* = 6.8 Hz, 2H, H_2_-2), 2.04 (s, 3H, CH_3_ Ph), 1.81 (m, 4H, H_2_-3 and H_2_-4), 1.71 (s, 6H, 2×CH_3_); ^13^C NMR (75 MHz, CDCl_3_) δ 173.9 (CO_2_CH_3_), 164.1 (CO), 159.2 (C-7), 158. 5 (C-8a), 157.4 (C-4), 154.8 (OC-2), 142.9 (C-6), 136.9 (C-5), 123.9 (C-1), 113.8 (CH-3), 110.6 (CH-8), 106.8 (C-2), 105.1 (CH-5), 101.2 (CH-6), 94.9 and 94.7 (2×OCH_2_O), 68.1 (CH_2_-5), 56.3 and 55.9 (2×OCH_3_), 51.8 (CO_2_CH_3_), 33.7 (CH_2_-2), 28.6 (CH_2_-4), 26.3 and 25.2 (2×CH_3_), 21.7 (CH_2_-3), 20.7 (CH_3_ Ph). HRMS (TOF, ES+) *m/z* calculated for C_27_H_34_O_10_ [M + H]^+^ 519.2225; found 519.2212.

#### 4.2.4. Preparation of methyl 5-((3,7-dihydroxy-1-methyl-6-oxo-6H-benzo[c]chromen-9-yl)oxy)pentanoate (**8**)

A 3 M solution of HCl in MeOH (150 µL, 0.450 mmol) was added to a solution of biaryl compound **7** (26.1 mg, 0.050 mmol) in anhydrous MeOH (1.5 mL) and the reaction mixture was stirred at rt for 22 h. After concentration under vacuum, the residue was dissolved in anhydrous CH_2_Cl_2_ (4 mL) and treated with trifluoroacetic acid (430 µL). Following stirring for 20 h at rt, thin layer chromatography showed the formation of a single compound and all the volatiles were removed under vacuum, using CHCl_3_ to co-evaporate the last traces of TFA. The obtained residue was purified by chromatography, using CHCl_3_ as eluent, to give benzochromenone derivative **8** (18.1 mg, 97%) as a white solid. ^1^H NMR (300 MHz, DMSO-*d*_6_) δ 11.79 and 10.33 (each s, 1H each, 2×OH), 7.13 (d, *J* = 2.2 Hz, 1H, H-10), 6.69 (d, *J* = 2.6 Hz, 1H, H-2), 6.61 (d, *J* = 2.6 Hz, 1H, H-4), 6.55 (d, *J* = 2.2 Hz, 1H, H-8), 4.11 (t, *J* = 5.9 Hz, 2H, H_2_-5), 3.59 (s, 3H, OCH_3_), 2.68 (s, 3H, CH_3_), 2.41 (t, *J* = 7.0 Hz, 2H, H_2_-2), 1.85–1.56 (m, 4H, H_2_-3 and H_2_-4); ^13^C NMR (75 MHz, DMSO-*d*_6_) δ 173.2 (CO_2_CH_3_), 165.5 (CO), 164.6 (C-9), 164.1 (C-7), 158.5 (C-3), 152.6 (C-4a), 138.4 (C-1), 137.7 (C-10a), 117.5 (CH_2_-2), 108.8 (C-10b), 103.6 (CH-10), 101.6 (CH-4), 99.5 (CH-8), 98.3 (C-6a), 67.8 (CH_2_-5), 51.2 (CO_2_CH_3_), 32.8 (CH_2_-2), 27.8 (CH_2_-4), 25.0 (CH_3_), 21.1 (C-3); HRMS (TOF, ES+) *m/z* calculated for C_20_H_21_O_7_ [M + H]^+^ 373.1282; found 373.1278.

#### 4.2.5. Preparation of 5-((3,7-dihydroxy-1-methyl-6-oxo-6H-benzo[c]chromen-9-yl)oxy)pentanoic acid (Hapten **AL*a***)

Lipase from *Candida antarctica* immobilized on acrylic resin (23 mg) was added to a solution of methyl ester **8** (16.6 mg, 0.0446 mmol) in a 4:1 mixture of 100 mM sodium phosphate buffer (pH 7.4) and THF (1.5 mL) at 30 °C. The resulting heterogeneous mixture was smoothly stirred for 24 h at rt and then filtered to separate the enzyme. The filtrated and washing THF phases were combined, diluted with EtOAc, washed with brine, dried over anhydrous MgSO_4_, and concentrated *in vacuo* to afford hapten **AL*a*** (14.9 mg, 93%) as a white amorphous solid. ^1^H NMR (300 MHz, THF-*d*_8_) δ 11.99 and 9.19 (each s, 1H each, 2×OH), 7.27 (d, *J* = 2.2 Hz, 1H, H-10), 6.67 (d, *J* = 2.7 Hz, 1H, H-2), 6.61 (d, *J* = 2.6 Hz, 1H, H-4), 6.56 (d, *J* = 2.2 Hz, 1H, H-8), 4.13 (t, *J* = 6.1 Hz, 2H, H_2_-5), 2.78 (s, 3H, CH_3_), 2.33 (t, *J* = 7.1 Hz, 2H, H_2_-2), 1.91–1.77 (m, 4H, H_2_-3 and H_2_-4); ^13^C NMR (126 MHz, THF-*d*_8_) δ 174.5 (CO_2_H), 167.1 (CO), 166.4 (C-9), 166.2 (C-7), 159.9 (C-3), 154.5 (C-4a), 139.5 (C-1), 139.2 (C-10a), 118.5 (CH-2), 110.7 (C-10b), 105.1 (CH-10), 102.8 (CH-4), 100.3 (CH-8), 100.0 (C-6a), 69.2 (CH_2_-5), 34.0 (CH_2_-2), 29.6 (CH_2_-4), 25.0 (CH_3_, overlapped with solvent signal), 22.6 (CH_2_-3); HRMS (TOF, ES+) *m/z* calculated for C_19_H_18_O_7_ [M + H]^+^ 359.1125; found 359.1122.

#### 4.2.6. Preparation of 2,5-dioxopyrrolidin-1-yl 5-((3,7-dihydroxy-1-methyl-6-oxo-6H-benzo[c]chromen-9-yl)oxy)pentanoate (**AL*a*-NHS** Ester)

A solution of hapten **AL*a*** (11.0 mg, 30.7 µmol), *N*-(3-dimethylaminopropyl)-*N*′-ethylcarbodiimide hydrochloride (EDC∙HCl) (7.0 mg, 36.8 µmol, 1.2 equiv) and *N*-hydroxisuccinimide (NHS) (5.0 mg, 43.4 µmol, 1.4 equiv) in anhydrous DMF (0.6 mL) was stirred at rt under nitrogen overnight. The reaction mixture was diluted with CH_2_Cl_2_, washed with water, a 1.5% (*w*/*v*) aqueous solution of LiCl and brine, dried over anhydrous MgSO_4_ and concentrated under reduced pressure to give the *N*-hydroxysuccinimidyl ester of hapten **AL*a****,* **AL*a*-NHS** ester, (13.8 mg, ca. 99% of crude product) as a slightly yellowish oil which was used immediately for the preparation of the corresponding protein bioconjugates. ^1^H NMR (500 MHz, THF-*d*_8_) δ 11.99 and 9.06 (each s, 1H each, 2×OH), 7.28 (d, *J* = 2.2 Hz, 1H, H-10), 6.66 (d, *J* = 2.6 Hz, 1H, H-2), 6.60 (d, *J* = 2.7 Hz, 1H, H-4), 6.57 (d, *J* = 2.2 Hz, 1H, H-8), 4.17 (t, *J* = 5.8 Hz, 2H, H_2_-5), 2.78 (s, 3H, CH_3_), 2.75 (br s, 4H, COCH_2_CH_2_CO), 2.72 (t, *J* = 7.0 Hz, 2H, H_2_-2), 1.95 (m, 4H, H_2_-3 and H_2_-4).

### 4.3. Synthesis of the N-hydroxysuccinimidyl Ester of Hapten **ALb**

#### 4.3.1. Preparation of 3,5-bis(benzyloxy)-2′,4′-bis(methoxymethoxy)-6′-methyl-[1,1′-biphenyl]-2-carbaldehyde (**10**)

An ampoule containing a mixture of freshly prepared aryl boronic acid **6** (104.5 mg, 0.408 mmol, 2 equiv), 2,4-*bis*(benzyloxy)-6-bromobenzaldehyde **9** (80.9 mg, 0.204 mmol), K_2_CO_3_ (63.6 mg, 0.460 mmol, 2.2 equiv) and Pd(PPh_3_)_4_ (26.6 mg, 0.023 mmol, 0.1 equiv) in anhydrous DMF (2 mL) was exhaustively degassed by freeze-thaw cycles. The ampoule was closed under vacuum and heated at 95 °C for 19 h. After cooling, the ampoule was opened and the reaction mixture was poured onto water and extracted with EtOAc. The combined organic extracts were washed with water, a 1.5% (*w*/*v*) aqueous solution of LiCl and brine, dried under anhydrous MgSO_4_ and concentrated under vacuum. The resulting crude reaction mixture was chromatographed on silica gel to give biaryl-2-carbaldehyde **10** (93.6 mg, 77%) as a viscous yellowish oil. ^1^H NMR (500 MHz, CDCl_3_) δ 10.02 (s, 1H, CHO), 7.54–7.48 (m, 2H, 2×CH Ph), 7.44–7.36 (m, 6H, 6xCH Ph), 7.36–7.29 (m, 2H, 2×CH Ph), 6.72 (d, *J* = 2.4 Hz, 1H, H-6), 6.65 (d, *J* = 2.3 Hz, 1H, H-4), 6.64 (d, *J* = 2.3 Hz, 1H, H-5′), 6.37 (d, *J* = 2.3 Hz, 1H, H-3′), 5.21–5.16 (m, two overlapped AB systems, 4H, OCH_2_O and OCH_2_Ph), 5.09 and 5.06 (AB system, *J* = 11.7 Hz, 1H each, OCH_2_Ph), 5.08 and 4.97 (AB system, *J* = 6.7 Hz, 1H each, OCH_2_O), 3.51 and 3.27 (each s, 3H each, 2×OCH_3_), 1.96 (s, 3H, CH_3_ Ph); ^13^C NMR (126 MHz, CDCl_3_) δ 189.6 (CHO), 163.5 (C-3), 162.1 (C-5), 157.6 (C-4′), 155.1 (C-2′), 144.8 (C-6′), 138.1 (C-1), 136.4 and 136.1 (2×C Ph), 128.9 (2×CH Ph), 128.8 (2×CH Ph), 128.4 (CH Ph), 128.1 CH Ph), 127.7 (2×CH Ph), 127.2 (2×CH Ph), 123.1 (C-1′), 118.6 (C-2), 110.7 (CH-5′), 109.6 (CH-3′), 101.2 (CH-6), 100.1 (CH-4), 94.7 and 94.6 (2×OCH_2_O), 70.7 and 70.4 (2×OCH_2_Ph), 56.3 and 56.1 (2×OCH_3_), 20.6 (CH_3_ Ph); HRMS (TOF, ES+) *m/z* calculated for C_32_H_33_O_7_ [M + H]^+^ 529.2221, found 529.2205.

#### 4.3.2. Preparation of 3,5-bis(benzyloxy)-2′,4′-bis(methoxymethoxy)-6′-methyl-[1,1′-biphenyl]-2-carboxylic Acid (**11**)

NaH_2_PO_4_·H_2_O (58.6 mg, 0.425 mmol, 2.8 equiv), 2-methylbut-2-ene (322.1 µL, 3.04 mmol, 20 equiv) and NaClO_2_ (45.3 mg, 0.501 mmol, 3.3 equiv) were successively added to a solution of biaryl-2-carbaldehyde **10** (80.4 mg, 0.152 mmol) in *^t^*BuOH (3.2 mL) and milli-Q water (0.4 mL) at 0 °C. The mixture was allowed to warm at rt and stirred for 5 h, then diluted with an aqueous saturated solution of NH_4_Cl and extracted with EtOAc. The combined organic layers were washed with brine and dried over anhydrous MgSO_4_. Chromatography on silica gel of the residue left after evaporation of the solvent at reduced pressure, using 8:2 hexane-EtOAc as eluent, gave the biaryl-2-carboxylic acid **11** (79.5 mg, 96%) as a semi solid. ^1^H NMR (500 MHz, CDCl_3_) δ 9.32 (s, 1H, CO_2_H), 7.61–7.32 (m, 10H, 10xCH Ph), 6.68 (d, *J* = 2.4 Hz, 2H, H-6 and H-4), 6.65 (d, *J* = 2.4 Hz, 1H, H-5′), 6.44 (d, *J* = 2.3 Hz, 1H, H-3′), 5.21–5.14 (m, two overlapped AB systems, 4H, OCH_2_O and OCH_2_Ph), 5.07 and 5.04 (AB system, *J* = 11.7 Hz, 1H each, OCH_2_Ph), 4.99 (br s, 2H, OCH_2_O), 3.50 and 3.16 (each s, 3H each, 2×OCH_3_), 2.01 (s, 3H, CH_3_ Ph); ^13^C NMR (126 MHz, CDCl_3_) δ 165.7 (CO_2_H), 161.1 (C-3), 157.8 (C-5), 157.6 (C-4′), 155.1 (C-2′), 141.3 (C-6′), 138.4 and 135.7 (2×C Ph), 128.9 (2×CH Ph), 128.8 (2×CH Ph), 128.6 (CH Ph), 128.4 (CH Ph), 127.7 (2×CH Ph), 127.6 (2×CH Ph), 125.1 (C-2), 115.9 (C-1′), 111.6 (CH-5′), 109.9 (CH-3′), 102.6 (CH-6), 100.3 (CH-4), 96.1 and 94.7 (2×OCH_2_O), 71.5 and 70.4 (2×OCH_2_Ph), 56.3 and 56.1 (2×OCH_3_), 20.5 (CH_3_ Ph); HRMS (TOF, ES+) *m/z* calculated for C_32_H_33_O_8_ [M + H]^+^ 545.2170, found 545.2156.

#### 4.3.3. Preparation of 7,9-bis(benzyloxy)-3-hydroxy-1-methyl-6H-benzo[c]chromen-6-one (**12**)

A 50:1 (*v*/*v*) mixture of ^i^PrOH and concentrated HCl (1.7 mL) was added to a solution of biaryl-2-carboxylic acid **11** (69.4 mg, 0.127 mmol) in THF (5.1 mL) at rt under nitrogen. The mixture was thermostated at 55 °C in an oil bath and stirred at this temperature for 24 h. After this time, the mixture was cooled to rt, diluted with a concentrated aqueous solution of NaHCO_3_ and extracted with Et_2_O. The organic phase was washed with brine, dried over anhydrous MgSO_4_ and concentrated under vacuum to give 7,9-*bis*(benzyloxy)alternariol **12** (54.7 mg, 98%) as an amorphous whitish solid. The crude reaction product thus obtained was sufficiently pure, as judged by its NMR spectroscopic data, to be used in the next step without further purification. ^1^H NMR (500 MHz, DMSO-*d*_6_) δ 7.58 (d, *J* = 7.4 Hz, 2H, 2×CH Ph), 7.48 (d, *J* = 7.1 Hz, 2H, 2×CH Ph), 7.44–7.31 (m, 6H, 6xCH Ph), 7.28 (d, *J* = 2.2 Hz, 1H, H-10), 6.90 (d, *J* = 2.2 Hz, 1H, H-8), 6.63 (d, *J* = 2.7 Hz, 1H, H-2), 6.53 (d, *J* = 2.7 Hz, 1H, H-4), 5.31 and 5.29 (each s, 2H each, 2×OCH_2_Ph), 2.63 (s, 3H, CH_3_ Ph); ^13^C NMR (126 MHz, DMSO-*d*_6_) δ 163.6 (CO), 162.4 (C-9), 158.4 (C-7), 156.5 (C-3), 153.7 (C-4a), 140.0 (C-1), 138.0 (C-10a), 136.8 and 136.3 (2×CH Ph), 128.7 (2×CH Ph), 128.5 (2×CH Ph), 128.3 (CH Ph), 127.9 (2×CH Ph), 127.7 (CH Ph), 127.0 (2×CH Ph), 116.7 (CH-2), 109.1 (C-10b), 103.2 (C-6a), 102.8 (CH-10), 100.9 (CH-4), 99.8 (CH-8), 70.1 and 69.9 (2×OCH_2_Ph), 25.0 (CH_3_ Ph); HRMS (TOF, ES+) *m/z* calculated for C_28_H_23_O_5_ [M + H]^·+^ 439.1540, found 439.1530.

#### 4.3.4. Preparation of methyl 5-((7,9-bis(benzyloxy)-1-methyl-6-oxo-6H-benzo[c]chromen-3-yl)oxy)pentanoate (**13**)

Methyl bromovalerate (29.5 mg, ca. 22 µL, 0.151 mmol, 1.1 equiv) was added via syringe to a stirred suspension of Cs_2_CO_3_ (57.8 mg, 0.177 mmol, 1.3 equiv) and phenol **12** (60.1 mg, 0.137 mmol) in anhydrous DMF (2 mL) at rt under nitrogen and the mixture was stirred for 19 h. The resulting pale yellowish reaction mixture was diluted with water and extracted with EtOAc. The combined organic extracts were washed successively with water, a 1.5% (*w*/*v*) aqueous solution of LiCl and brine, dried over anhydrous MgSO_4_ and concentrated under reduced pressure. The crude reaction product was purified by chromatography on silica gel, using CHCl_3_ as eluent, to afford the *O*-alkylated product **13** (71.4 mg, 94%) as a pale yellowish semi-solid. ^1^H NMR (500 MHz, CDCl_3_) δ 7.59 (m, 2H, CH Ph), 7.43–7.34 (m, 8H, 8×CH Ph), 7.30 (d, *J* = 2.3 Hz, 1H, H-2), 6.67 (d, *J* = 2.7 Hz, 1H, H-10), 6.64 (d, *J* = 4.6 Hz, 2H, H-4), 6.64 (s, 1H, H-8), 5.28 and 5.16 (each s, 2H each, 2×OCH_2_Ph), 4.00 (t, *J* = 5.5 Hz, 2H, H_2_-5), 3.68 (s, 3H, CO_2_CH_3_), 2.67 (s, 3H, CH_3_), 2.41 (t, *J* = 6.9 Hz, 2H, H_2_-2), 1.84 (m, 4H, H_2_-3 and H_2_-4); ^13^C NMR (126 MHz, CDCl_3_) δ 173.9 (CO_2_CH_3_), 163.7 (CO), 162.9 (C-9), 159.5 (C-7), 157.8 (C-3), 154.3 (C-4a), 140.7 (C-1), 137.4 (C-10a), 136.5 and 135.9 (2×C Ph), 129.0 (2×CH Ph), 128.8 (2×CH Ph), 128.6 (CH Ph), 127.9 (CH Ph), 127.5 (2×CH Ph), 126.8 (2×CH Ph), 116.7 (CH-2), 111.0 (C-10b), 104.5 (C-6a), 103.9 (CH-10), 100.0 (CH-4), 99.9 (CH-8), 71.0 and 70.5 (each OCH_2_Ph), 67.7 (CH_2_-5), 51.7 (OCH_3_), 33.8 (CH_2_-2), 28.6 (CH_2_-4), 25.6 (CH_3_), 21.7 (CH_2_-3); HRMS (TOF, ES+) *m/z* calculated for C_34_H_33_O_7_ [M + H]^+^ 553.2221, found 553.2112.

#### 4.3.5. Preparation of 5-((7,9-bis(benzyloxy)-1-methyl-6-oxo-6H-benzo[c]chromen-3-yl)oxy)pentanoic Acid (**14**)

The hydrolysis of the methyl ester moiety of **13** was performed following the same procedure reported for the hydrolysis of ester **8** to obtain hapten **AL*a***. The methyl ester **13** (17.5 mg, 0.032 mmol), lipase from *Candida antarctica* immobilized on acrylic resin (16 mg) and a 4:1 mixture of 100 mM sodium phosphate buffer (pH 7.4) and THF (1.1 mL). Workup as described for the hydrolysis of **8** yielded acid **14** (17.0 mg, 99%) as a whitish semi-solid. ^1^H NMR (500 MHz, THF-*d*_8_) δ 7.68 (m, 2H, 2×CH Ph), 7.46 (m, 2H, 2×CH Ph), 7.40–7.30 (m, 6H, 4×CH Ph and H-2), 7.25 (br t, *J* = 7.5 Hz, 1H, CH Ph), 6.85 (d, *J* = 2.2 Hz, 1H, H-10), 6.71 (d, *J* = 2.8 Hz, 1H, H-4), 6.69 (d, *J* = 2.8 Hz, 1H, H-8), 5.26 (s, 4H, 2×OCH_2_Ph), 4.05 (t, *J* = 6.2 Hz, 2H, H_2_-5), 2.71 (s, 3H, Ar-CH_3_), 2.32 (t, *J* = 7.2 Hz, 2H, H_2_-2), 1.78 (m, 4H, H_2_-3 and H_2_-4). ^13^C NMR (126 MHz, THF-*d*_8_) δ 174.5 (CO_2_H), 164.8 (CO), 163.9 (C-9), 160.9 (C-7), 156.7 (C-3), 155.7 (C-4a), 141.4 (C-1), 138.5 (C-10a), 138.3 and 137.9 (each C Ph), 129.5 (2×CH Ph), 129.2 (2×CH Ph), 129.0 (CH Ph), 128.5 (2×CH Ph), 128.2 (CH Ph), 127.6 (2×CH Ph), 117.2 (CH-2), 111.8 (C-10b), 105.4 (C-6a), 104.5 (CH-10), 100.7 (CH-4), 100.5 (CH-8), 71.4 and 71.1 (each OCH_2_Ph), 68.8 (CH_2_-5), 34.0 (CH_2_-2), 29.7 (CH_2_-4), 26.5 (CH_3_), 22.6 (CH_2_-3); HRMS (TOF, ES+) *m/z* calculated for C_33_H_31_O_7_ [M + H]^+^ 539.2064, found 539.2073.

#### 4.3.6. Preparation of 2,5-dioxopyrrolidin-1-yl 5-((7,9-dihydroxy-1-methyl-6-oxo-6H-benzo[c]chromen-3-yl)oxy)pentanoate (**AL*b*-NHS** Ester)

The acid **14** obtained in the above step (15.1 mg, 28 µmol) was transformed into the corresponding *N*-hydroxysuccinimidyl ester **15** (17.2 mg) following the same procedure previously described for the transformation of hapten **AL*a*** into **AL*a*-NHS** ester, using EDC∙HCl (6.4 mg, 33.6 µmol, 1.2 equiv) and NHS (4.2 mg, 36.5 µmol, 1.3 equiv) in anhydrous DMF (1 mL). ^1^H NMR (300 MHz, CDCl_3_) δ 7.59 (m, 2H, 2×CH Ph), 7.45–7.32 (m, 8H, 8xCH Ph), 7.31 (d, *J* = 2.3 Hz, 1H, H-2), 6.69 (d, *J* = 2.7 Hz, 1H, H-10), 6.67 (d, *J* = 2.7 Hz, 1H, H-4), 6.65 (d, *J* = 2.2 Hz, 1H, H-8), 5.29 and 5.17 (each s, 2H each, 2×OCH_2_Ph), 4.04 (t, *J* = 5.6 Hz, 2H, H_2_-5), 2.85 (br s, 4H, COCH_2_CH_2_CO), 2.72 (t, *J* = 6.9 Hz, 1H, H_2_-2), 2.68 (s, 3H, CH_3_), 1.96 (m, 4H, H_2_-3 and H_2_-4).

Thereafter, a suspension of 5% Pd/C (8 mg) and **15** in acetone (3 mL) was degassed and purged with hydrogen by several cycles of freeze-pump-thaw using a water aspirator pump. The hydrogen pressure was adjusted to 1.5 atm and the mixture was stirred vigorously overnight at rt. The reaction mixture was filtered through a disposable Teflon membrane filter (0.45 µm), and the filtrate and washing THF phases were combined and concentrated at reduced pressure to give the *N*-hydroxysuccinimidyl ester of hapten **AL*b***, **AL*b*-NHS** ester, (12.2 mg, 95% of crude product from **14**) as a viscous colorless oil which was used immediately for the preparation of the corresponding protein bioconjugates. ^1^H NMR (500 MHz, THF-*d*_8_/DMSO-d_6_) δ 11.92 and 9.67 (each s, 1H each, 2×OH), 7.24 (d, *J* = 2.1 Hz, 1H, H-2), 6.83 (d, *J* = 2.7 Hz, 1H, H-4), 6.82 (d, *J* = 2.6 Hz, 1H, H-10), 6.36 (d, *J* = 2.1 Hz, 1H, H-8), 4.10 (t, *J* = 5.6 Hz, 1H, H_2_-5), 2.79 (s, 3H, CH_3_), 2.76 (br s, 4H, COCH_2_CH_2_CO), 2.71 (t, *J* = 6.9 Hz, 2H, H_2_-2), 1.93 (m, 4H, H_2_-3 and H_2_-4).

### 4.4. Immunoreagent Preparation

Protein conjugates of the two haptens were obtained by the active ester method. A 50 mM solution of the activated hapten was prepared in DMSO. BSA and OVA solutions were prepared at 15 mg/mL in 50 mM carbonate buffer, pH 9.6. The activated hapten was added to the protein solution at 40-fold molar excess for BSA conjugates. Conjugation reactions to OVA were done with 8- or 11-fold excess for **AL*a*** and **AL*b***, respectively. Concerning HRP conjugates, the activated hapten solution (5 mM) was added over a 3 mg/mL enzyme solution in the same carbonate buffer to reach a hapten molar excess of 15. The activated haptens were added slowly to the protein solution (ca. 15–20 μL per hour), and the mixtures were incubated overnight at rt, protected from light, and with gentle stirring. Then, they were centrifuged for 5 min at 6700× *g*, and the conjugates were purified from the supernatant by size-exclusion chromatography using 100 mM phosphate buffer, pH 7.4, as eluent. Fractions containing BSA or OVA conjugates were pooled and diluted with elution buffer to a final concentration of 1 mg/mL. BSA conjugate solutions were passed through 0.45 μm sterile filters. BSA and OVA conjugate solutions were stored at −20 °C. HRP conjugate solutions were 1:1 diluted with PBS containing 1% BSA (*w*/*v*) and 0.02% (*w*/*v*) thimerosal and stored at 4 °C. The hapten-to-protein molar ratio of the prepared conjugates was determined by MALDI-TOF-MS and running BSA, OVA, and HRP for reference in the same plate, as previously described [27].

Animal manipulation was performed according to Spanish laws (RD1201/2005 and law 32/2007) and the European Directive 2010/63EU regarding the protection of experimental animals. Polyclonal antibodies to AOH and AME were obtained from the sera of immunized animals. Briefly, two female New Zealand white rabbits—weighing 2 kg at the beginning of the experiment—were immunized by four periodic subcutaneous injections of a 1:1 water-in-oil emulsion containing 300 μg of the BSA-hapten conjugate. The inoculum was prepared with complete Freund’s adjuvant for the first injection and with incomplete Freund’s adjuvant for subsequent injections. Boosts were applied with 21-day intervals. Animals were exsanguinated by intracardiac puncture 10 days after the last injection, and the blood was left overnight in the refrigerator at 4 °C for coagulation. Sera were separated from cells by centrifugation (3000× *g*, 20 min). Finally, immunoglobulins were partially purified by precipitation twice with one volume of cold saturated (3.9 M) ammonium sulphate solution. Antibodies were stored at 4 °C as precipitates.

### 4.5. Competitive ELISA Procedures

Immunoassays were carried out by competitive ELISA using the capture antibody-coated direct format and the conjugate-coated indirect format. After each incubation step, plates were washed three times with a 150 mM NaCl solution containing 0.05% (*v*/*v*) Tween 20. For direct assays, microplates were coated by overnight incubation at 4 °C with 100 μL per well of GAR solution (1 μg/mL) in 50 mM carbonate-bicarbonate buffer, pH 9.6. For indirect assays, microwells were coated with 100 μL per well of OVA-hapten conjugate solution in the same coating buffer, and overnight incubation at rt. The competitive reaction was performed by mixing in each well 50 μL of analyte solution in PBS with 50 μL of antibody dilution or enzyme tracer solution in PBS containing 0.05% (*v*/*v*) of Tween-20, and incubating 1 h at rt. For indirect assays, 100 μL per well of GAR-HRP diluted 1/10,000 in PBS containing 0.05% (*v*/*v*) of Tween-20 and 10% (*v*/*v*) of adult bovine serum was added. Signal was obtained using 100 μL per well of TMB as the chromogenic enzyme substrate and incubation at rt during 10 min. Finally, 100 μL of 1 M H_2_SO_4_ was added and the absorbance was read at 450 nm using 650 nm as reference wavelength.

Standard mycotoxin solutions were obtained by serially diluting in buffer the most concentrated standard solution, which was prepared from a concentrated stock solution in DMF. Eight-point standard curves were built using those solutions and a blank sample. SigmaPlot software, version 14.0 from Systat Software Inc. (San Jose, CA, USA), was employed to fit the experimental values to a standard four-parameter logistic equation. The half-maximal inhibition concentration (IC_50_) and the maximum absorbance (A_max_) values were considered in order to compare antibody performance. CR was calculated according to Formula (1):

CR (%) = IC_50_ (AOH)/IC_50_ (AME) × 100 (1)

## Figures and Tables

**Table 1:** Antibody characterization by checkerboard direct and indirect competitive ELISA using the corresponding homologous conjugate (n = 3) ^a^.

	Assay Conjugate									
	Direct Assay					Indirect Assay				
pAb	[pAb] ^b^	[HRP] ^c^	IC_50_^d^ AOH	IC_50_ AME	CR ^e^ (%)	[pAb]	[OVA]	IC_50_ AOH	IC_50_ AME	CR (%)
AL*a*#1	10	10	2.20	2.20	100	100	100	6.28	3.08	204
AL*a*#2	10	10	7.61	3.83	199	10	10	36.2	8.18	442
										
AL*b*#1	10	10	1.19	170	0.70	100	100	6.23	402	1.55
AL*b*#2	10	10	1.19	218	0.55	300	100	27.5	403	6.82

^a^ The A_max_ values were higher than 1.0. ^b^ Dilution factor × 10^−3^. ^c^ Bioconjugate concentration in ng/mL. ^d^ Values are in nM. ^e^ Cross-reactivity values with AOH as reference.

**Table 2:** Antibody characterization by checkerboard indirect competitive ELISA using the corresponding heterologous coating conjugate (n = 3) ^a^.

pAb	[pAb] ^b^	[OVA] ^c^	IC_50_^d^ AOH	IC_50_ AME	CR (%)
AL*a*#1	20	100	3.83	6.06	63.2
AL*a*#2	10	100	11.2	2.80	400
					
AL*b*#1	20	1000	3.32	230	1.44
AL*b*#2	30	1000	13.4	168	7.98

^a^ The A_max_ values were higher than 1.0. ^b^ Dilution factor × 10^−3^. ^c^ Bioconjugate concentration in ng/mL. ^d^ Values are in nM.

## Data Availability

Not applicable.

## Supplementary Materials

The following are available online at https://www.mdpi.com/2072-6651/13/12/883/s1: General procedures, materials, and equipment; NMR spectra of all of the intermediates and the final activated haptens.
