# Correction: Addante-Moya et al. Assessment of the Optimum Linker Tethering Site of Alternariol Haptens for Antibody Generation and Immunoassay Development. *Toxins* 2021, *13*, 883

**DOI:** 10.3390/toxins15020162

**Published:** 2023-02-16

**Authors:** Luis G. Addante-Moya, Antonio Abad-Somovilla, Antonio Abad-Fuentes, Consuelo Agulló, Josep V. Mercader

**Affiliations:** 1 Department of Organic Chemistry, University of Valencia, Doctor Moliner 50, 46100 Burjassot, Valencia, Spain; 2 Spanish Council for Scientific Research, Institute of Agrochemistry and Food Technology, Agustí Escardino 7, 46980 Paterna, Valencia, Spain

## Misplace of Schemes

In the original publication [1], Scheme 1 and Scheme 2 are misplaced due to mistakes in the publication process. Scheme 1 should be placed in the position of Scheme 2 and vice versa. 

The correct schemes should be as follows:

**Scheme 1:**
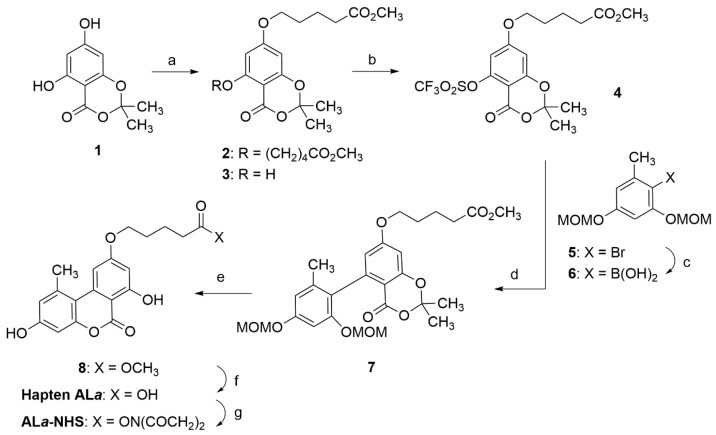
Synthesis of **AL*a*-NHS** ester. Reagents and conditions: (a) Br(CH_2_)_4_CO_2_CH_3_, K_2_CO_3_, KI, Bu_4_NBr, acetone, reflux, 16 h, 75% of **3**. (b) Tf_2_O, pyridine, 0 °C to rt, 20 h, 91%. (c) i. n-BuLi, THF, −78 °C, 40 min; ii. B(O^i^Pr)_3_, −78 °C to 0 °C, 1.5 h, 93%. (d) Pd(PPh_3_)_4_, K_2_CO_3_, DMF, 93 °C, 24 h, 75%. (e) i. HCl, MeOH, rt, 22 h; ii. TFA, CH_2_Cl_2_, rt, 20 h, 97%. (f) Lipase acrylic resin, THF-PB 100 mM, rt, 20 h, 93%. (g) EDC·HCl, NHS, DMF, rt, overnight, 99% of crude product.

**Scheme 2:**
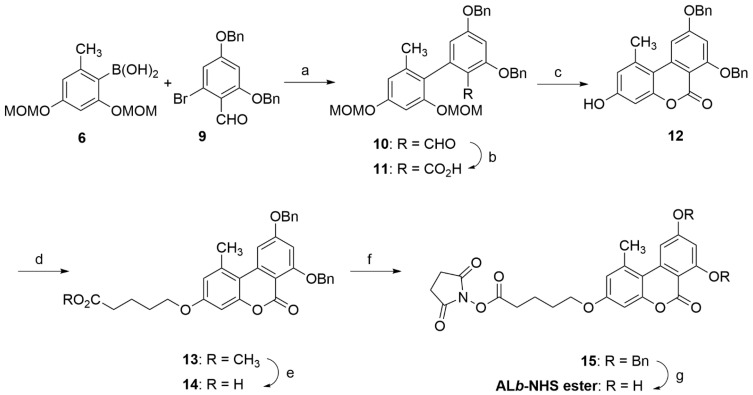
Synthesis of **AL*b*-NHS** ester. Reagents and conditions: (a) Pd(PPh_3_)_4_, K_2_CO_3_, DMF, 95 °C, 19 h, 77%. (b) NaH_2_PO_4_·H_2_O, NaClO_2_, ^t^BuOH-H_2_O (5:1), rt, 5 h, 96%. (c) ^i^PrOH, THF, conc HCl, 55 °C, 24 h, 98%. (d) Br(CH_2_)_4_CO_2_CH_3_, Cs_2_CO_3_, DMF, 94%. (e) Lipase acrylic resin, THF-PB 100 mM, rt, 20 h, 99%. (f) EDC·HCl, NHS, DMF, rt, overnight. (g) 5% Pd/C, acetone, H_2_ (1.5 atm), rt, 19 h, 95% of crude product from **14**.

The authors state that the scientific conclusions are unaffected. The original publication has also been updated.
