# Supplementary material for: Assessment of the Optimum Linker Tethering Site of Alternariol Haptens for Antibody Generation and Immunoassay Development
Source: Toxins (Basel). 2021 Dec 10;13(12):883. doi: 10.3390/toxins13120883 (PMC8705777; doi:10.3390/toxins13120883)

Supplementary Materials

Assessment of the optimum linker tethering site of alternariol haptens for antibody generation and immunoassay development

General

All solvents were purified by distillation and, if necessary, dried using conventional procedures.[1] All air-sensitive compound reactions were carried out in oven-dried glassware in a nitrogen atmosphere. Thin-layer chromatography (TLC) with 0.25 mm pre-coated silica gel plates was used to monitor the reactions. UV light and an aqueous ceric ammonium molybdate solution were used to visualize the results. Chromatography refers to flash column chromatography, which was performed on silica gel 60 (particle size 0.040–0.063 mm) with the given solvents. A Nicolet Avatar 320 FT-IR spectrophotometer with ATR was used to acquire IR spectra (IR band intensities: w = weak, m = medium, s = strong). $^{1}H/^{13}C$ NMR spectra were recorded at 298 ^{o}K, in the solvent indicated, at 300/75 MHz (Bruker Avance DPX300 spectrometer) or 500/126 MHz (Bruker Avance DRX500). In all cases, the chemical shifts are given in ppm (δ scale) relative to the residual solvent as the internal reference [7.27/77.00 ppm, 3.58/67.57, and 2.50/39.52 ppm, respectively, for the $^{1}H/^{13}C$ spectra in $CDCl_3$, THF-d_8 and DMSOd_6. DEPT pulse sequences were used to determine carbon substitution degrees. A combination of COSY and HSQC experiments

[1] Perrin, D. D; Armarego, W. L .F., in "Purification of Laboratory Chemicals", 4th ed.; Butterworth Heinemann Press: Oxford, 1996.

were used to assign the ^{1}H and ^{13}C chemical shifts of selected compounds. High resolution mass spectra (HRMS) were acquired utilizing the electrospray (ES) ionization mode on a Q-TOF premier mass spectrometer with an electrospray source (Waters, Manchester, UK).

Sephadex G-25 HiTrap® Desalting columns for protein–hapten conjugate purification and Sepharose HiTrap® Protein G HP columns for antibody purification were obtained from GE Healthcare (Uppsala, Sweeden) and operated under an ÄKTA Purifier workstation also from GE Healthcare. A 5800 matrix-assisted laser desorption ionization time-of-flight (MALDI-TOF/TOF) MS apparatus from AB Sciex (Framingham, MA) was used for bioconjugate analysis. ELISA tests were carried out with Costar® 96-well flat-bottom high-binding polystyrene ELISA plates from Corning (Corning, NY, USA). Microplate wells were washed with an ELx405 washer from BioTek Instruments (Winooski, VT, USA). Immunoassay absorbance values were read with a PowerWave HT microplate reader also from BioTek.

Copies of ^{1}H and ^{13}C NMR spectra

^{1}H NMR spectrum (300 MHz) of phenol **3** in $CDCl_3$

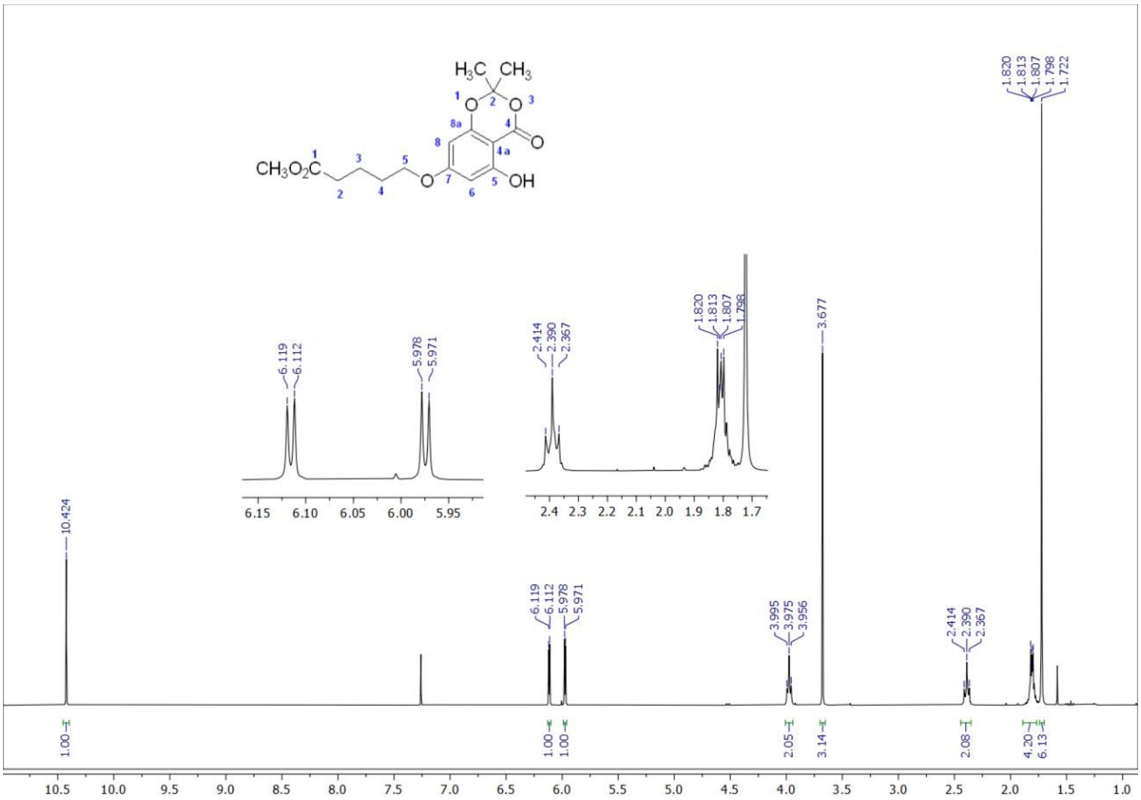

^{13}C NMR spectrum (75 MHz) of phenol **3**in $CDCl_3$

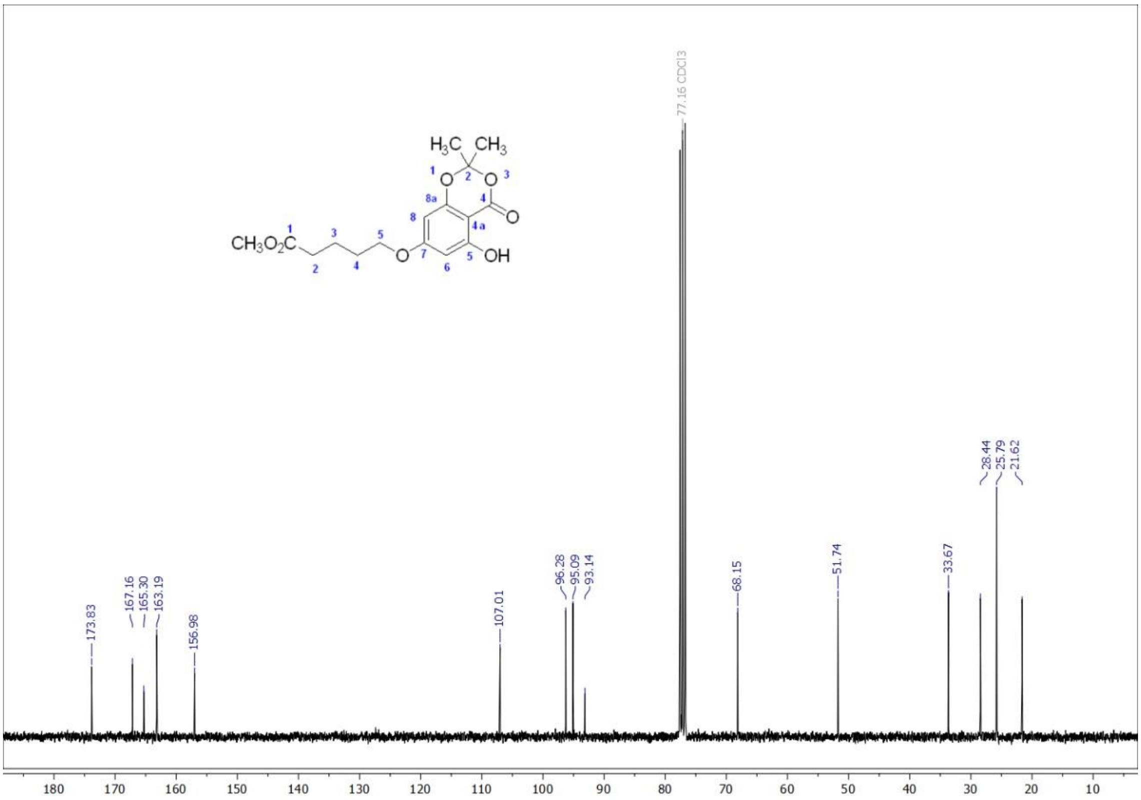

^{1}H NMR spectrum (300 MHz) of aryl triflate **4** in $CDCl_3$

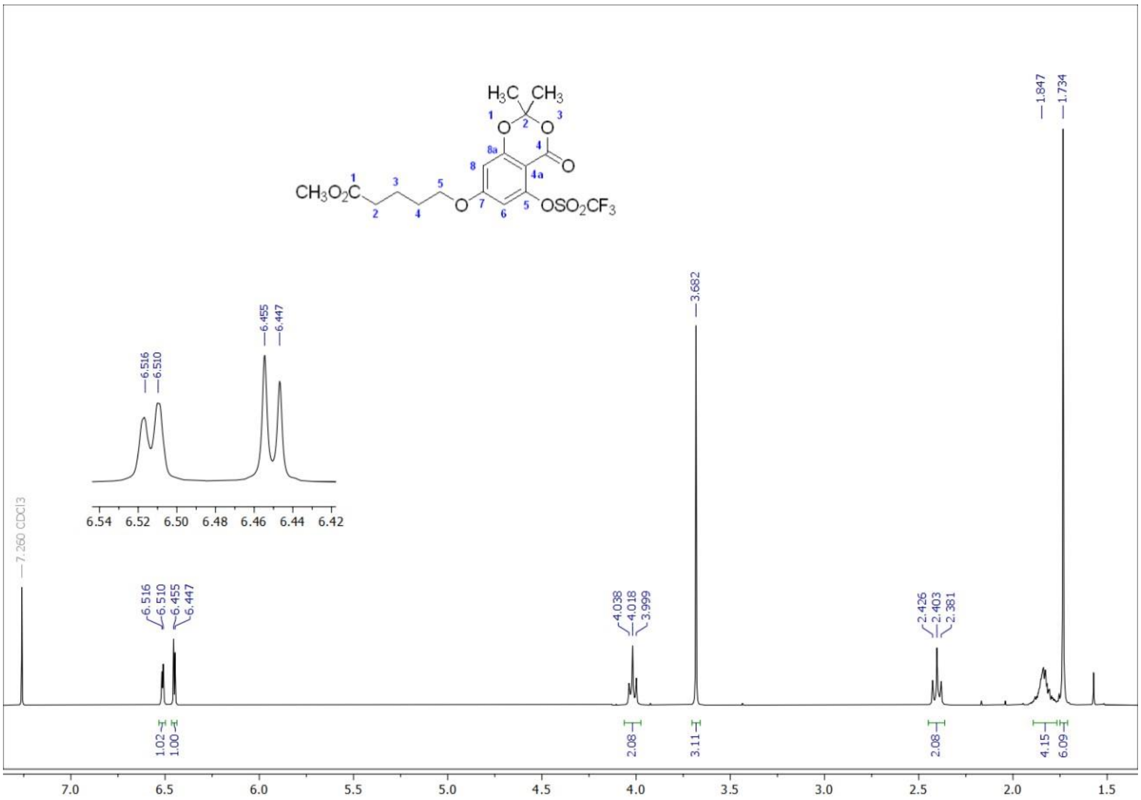

^{13}C NMR spectrum (75 MHz) of aryl triflate **4** in $CDCl_3$

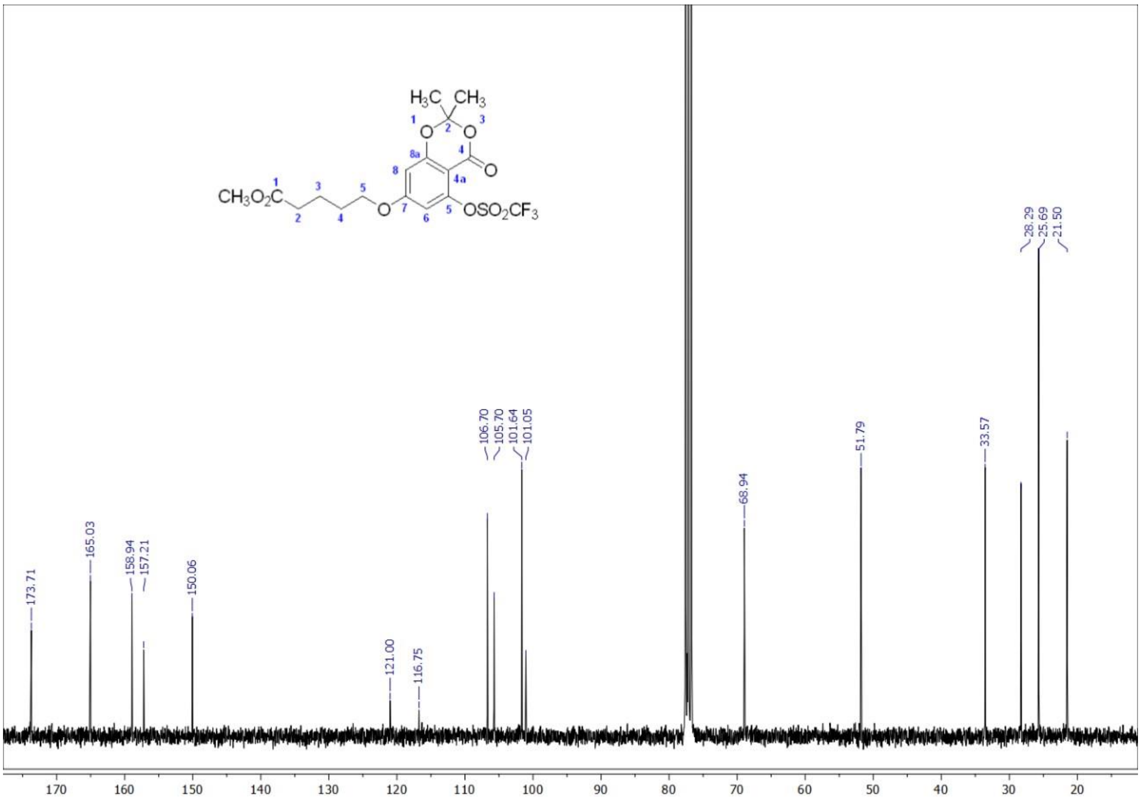

^{1}H NMR spectrum (300 MHz) of aryl boronic acid **6** in DMSO-d_6

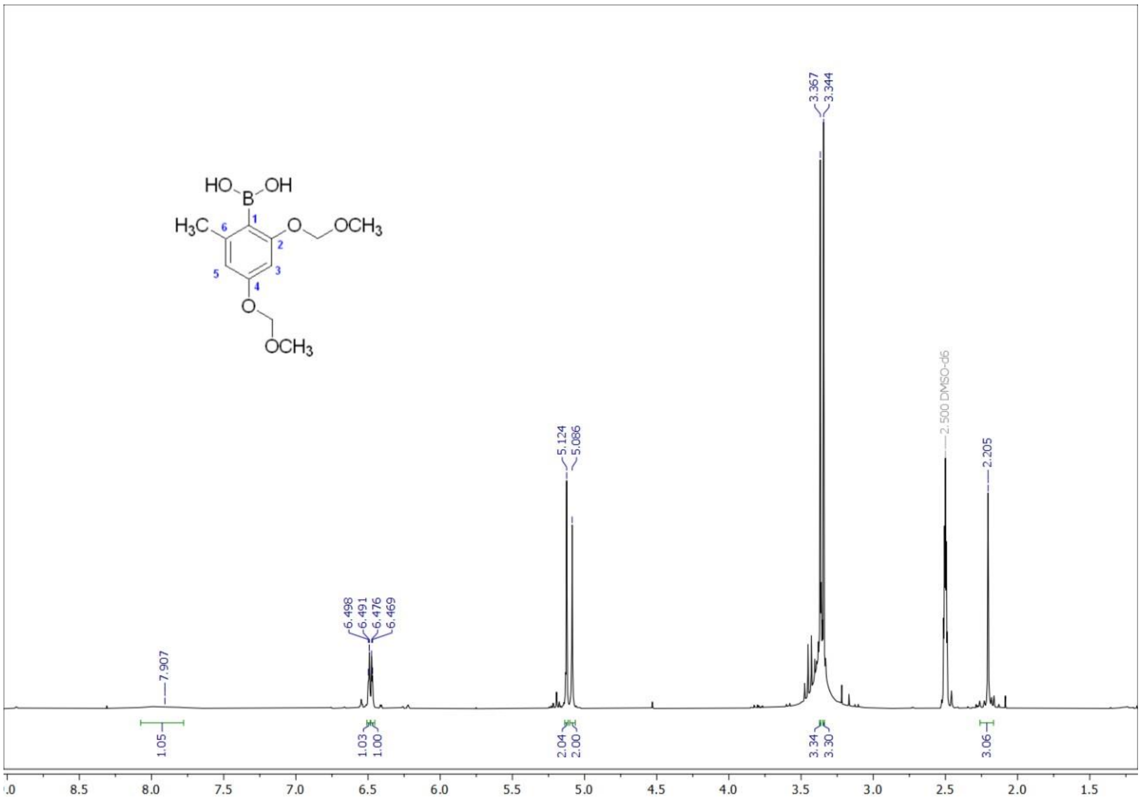

HO
B
OH
H3C
O
OCH3
O
OCH3
1
2
3
4
5
6
7.907
6.498
6.491
6.476
6.469
5.124
5.086
3.367
3.344
2.500 DMSO-d6
2.205
1.05
1.03
1.00
2.04
2.00
3.34
3.30
3.06
8.5
8.0
7.5
7.0
6.5
6.0
5.5
5.0
4.5
4.0
3.5
3.0
2.5
2.0
1.5

^{1}H NMR spectrum (300 MHz) of biaryl compound **7** in $CDCl_3$

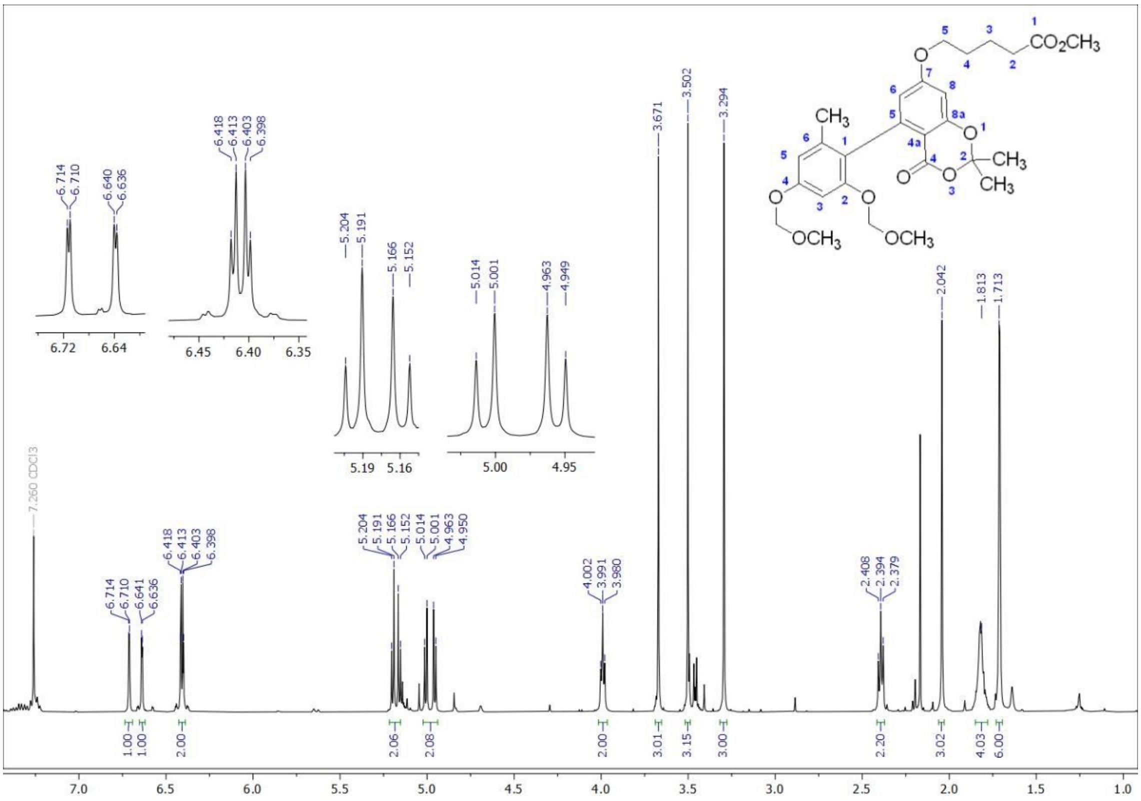

^{13}C NMR spectrum (75 MHz) of biaryl compound **7** in $CDCl_3$

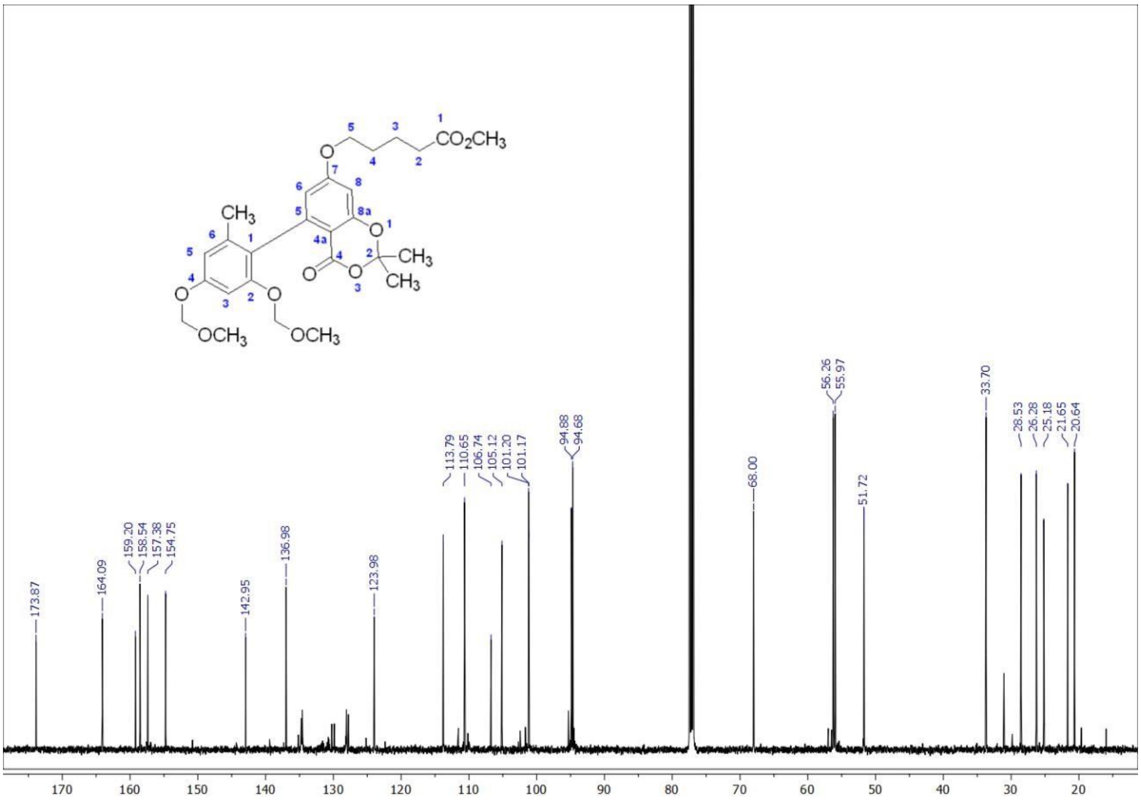

^{1}H NMR spectrum (300 MHz) of compound **8** in DMSO-d_6

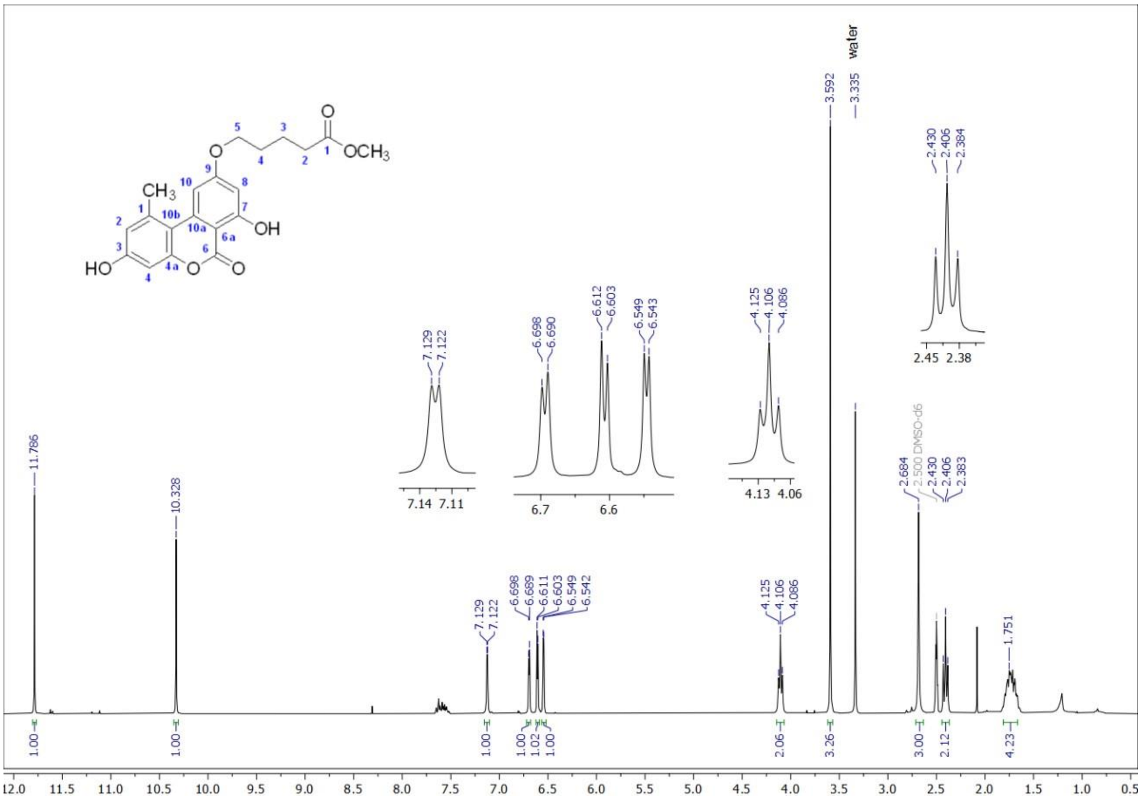

^{13}C NMR spectrum (75 MHz) of compound **8**in DMSO-d_6

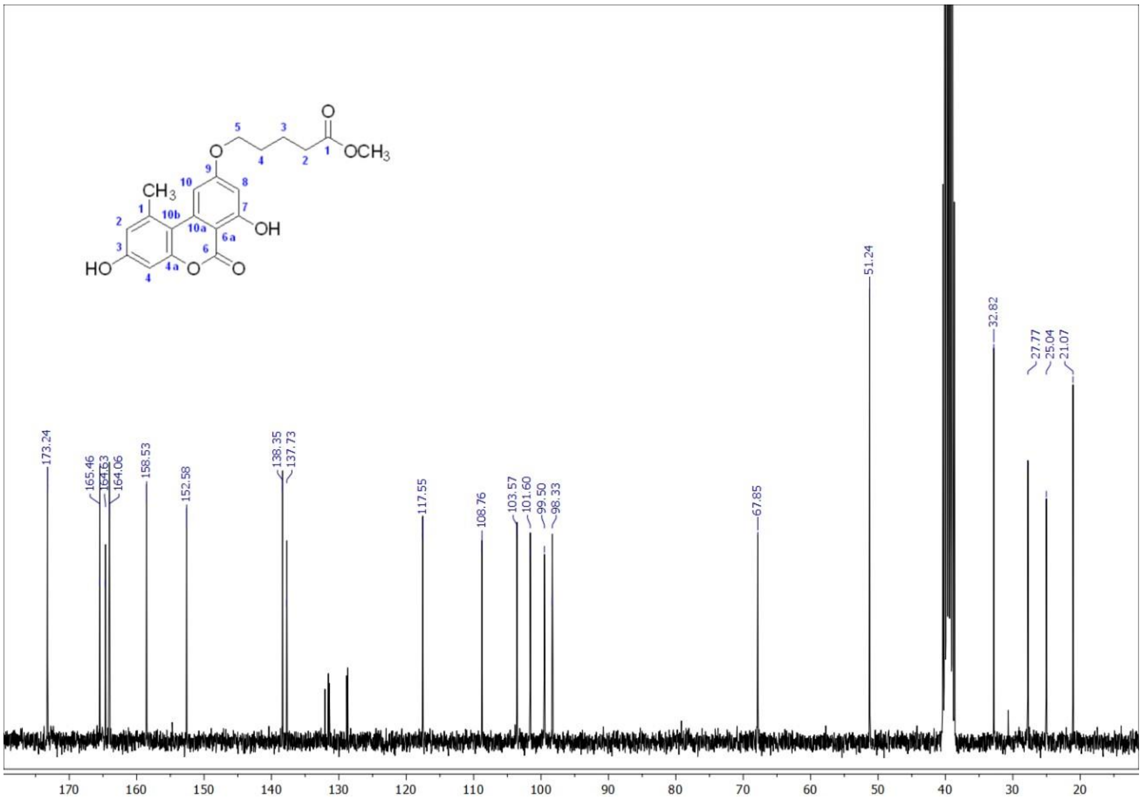

^{1}H NMR spectrum (300 MHz) of hapten AL*a* in THF-d_8

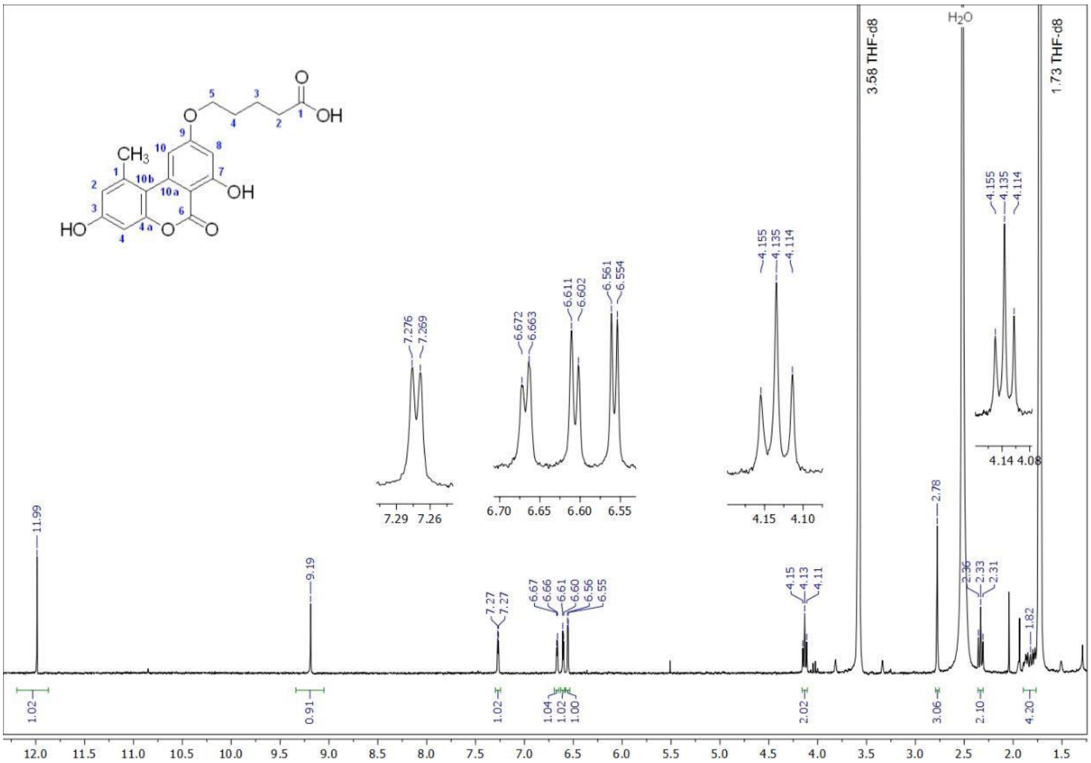

^{13}C NMR spectrum (126 MHz) of hapten AL*a* in THF-d_8

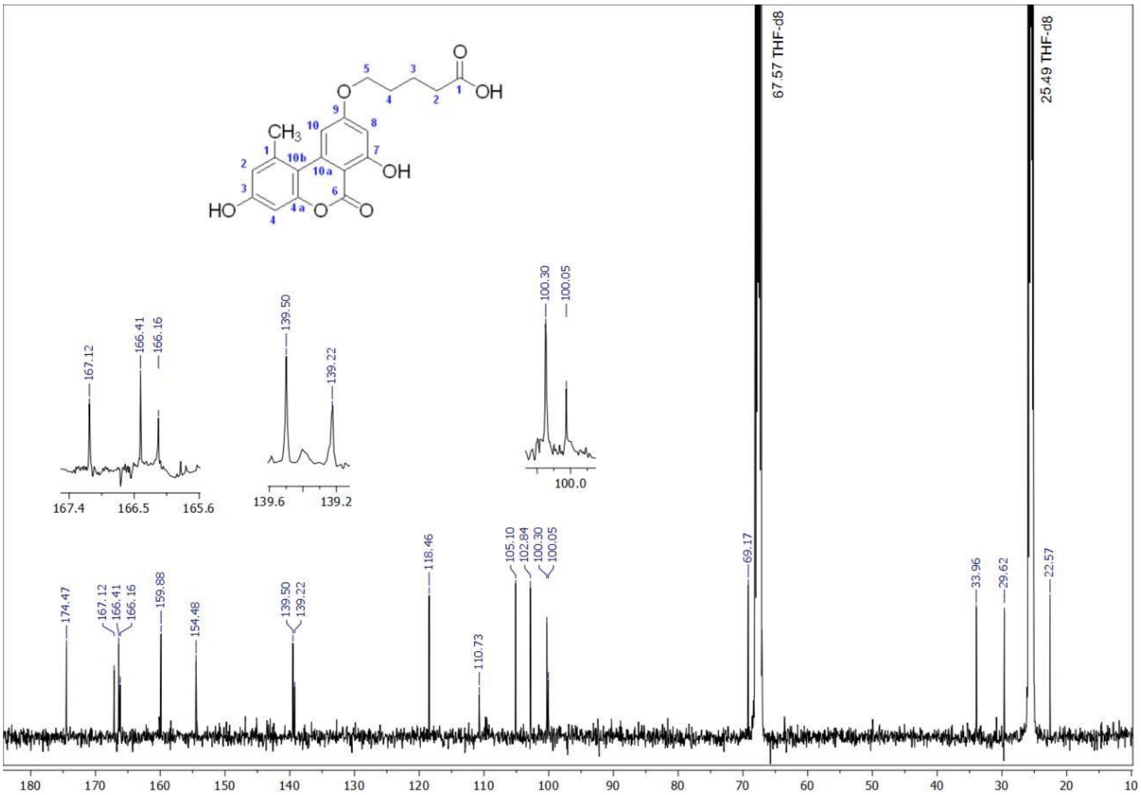

^{1}H NMR spectrum (500 MHz) of AL*a*-NHS ester in THF-d_8

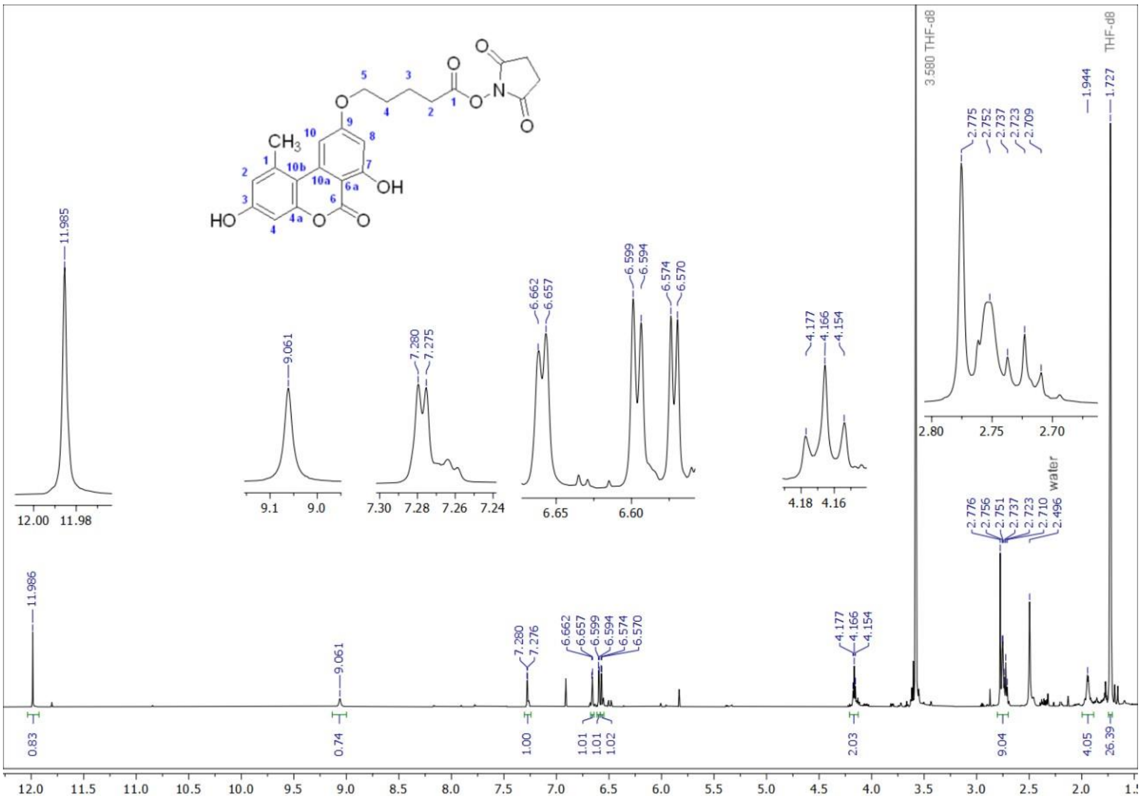

^{1}H NMR spectrum (500 MHz) of biphenyl-2-carbaldehyde **10** in $CDCl_3$

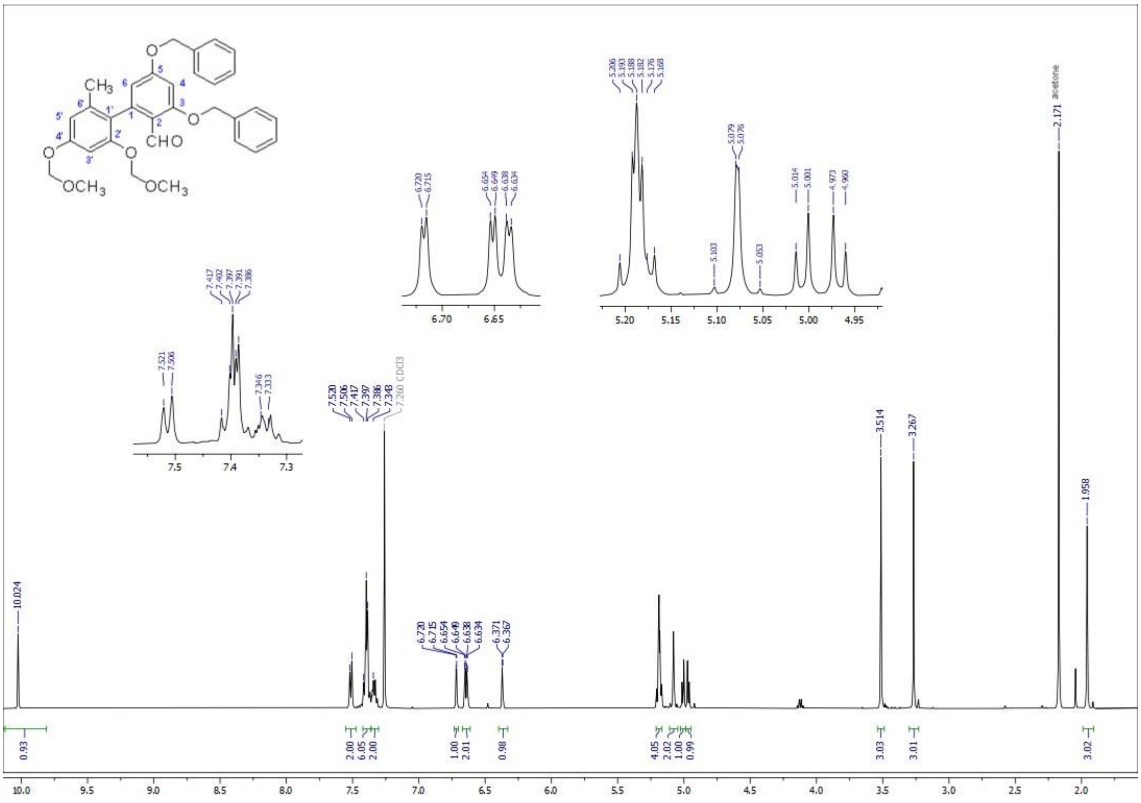

^{13}C NMR spectrum (126 MHz) of biphenyl-2-carbaldehyde **10** in $CDCl_3$

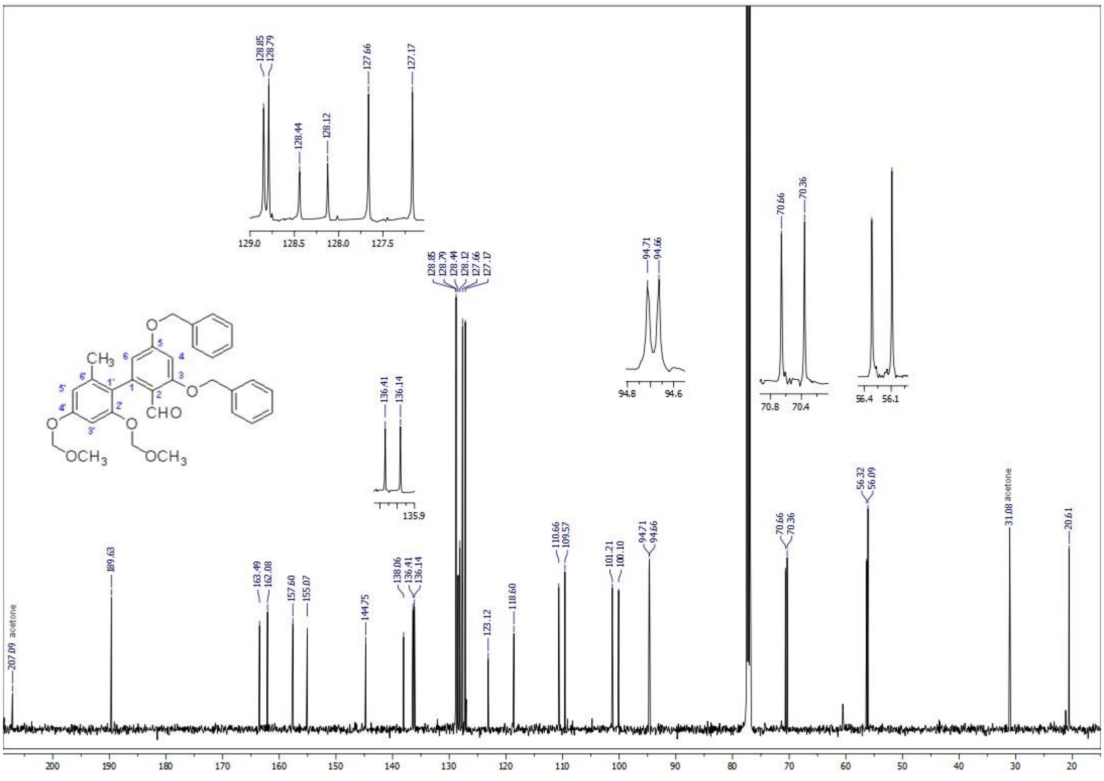

^{1}H NMR spectrum (500 MHz) of biphenyl-2-carboxylic acid **11** in $CDCl_3$

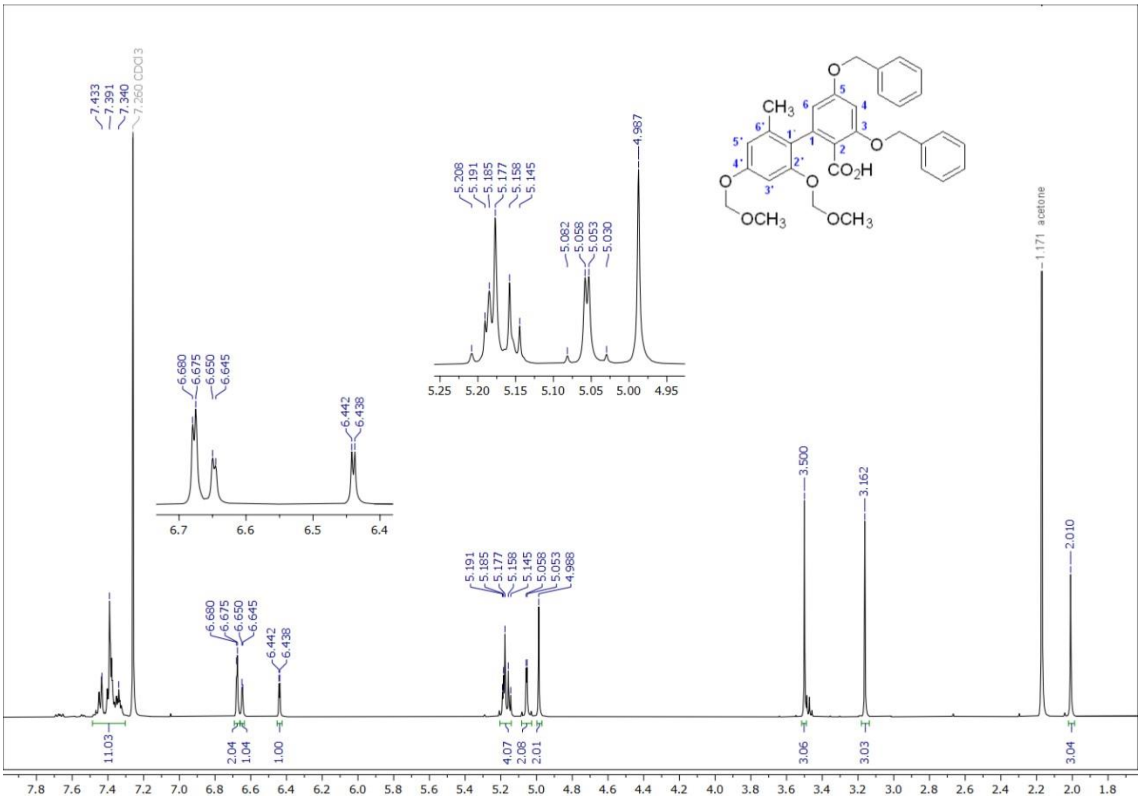

^{13}C NMR spectrum (126 MHz) of biphenyl-2-carboxylic acid **11** in $CDCl_3$

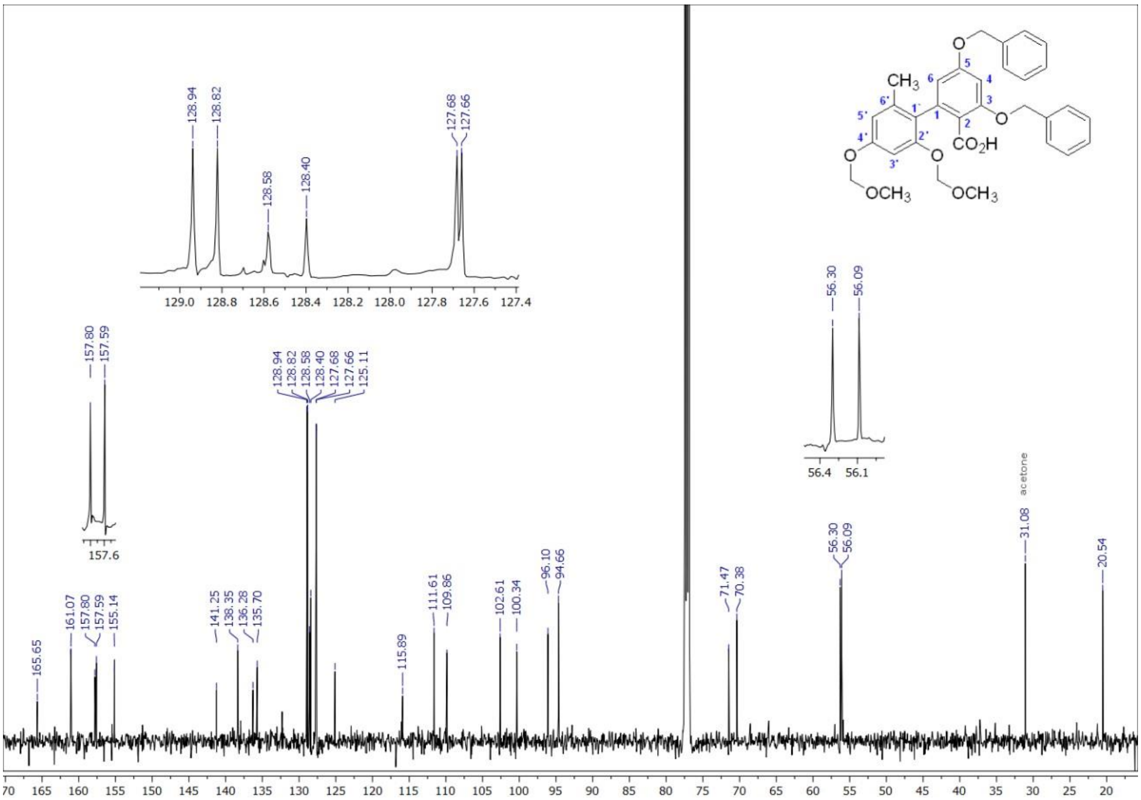

^{1}H NMR spectrum (500 MHz) of 7,9-dibenzyl ether of alternariol (**12**) in DMSO-d$_6$

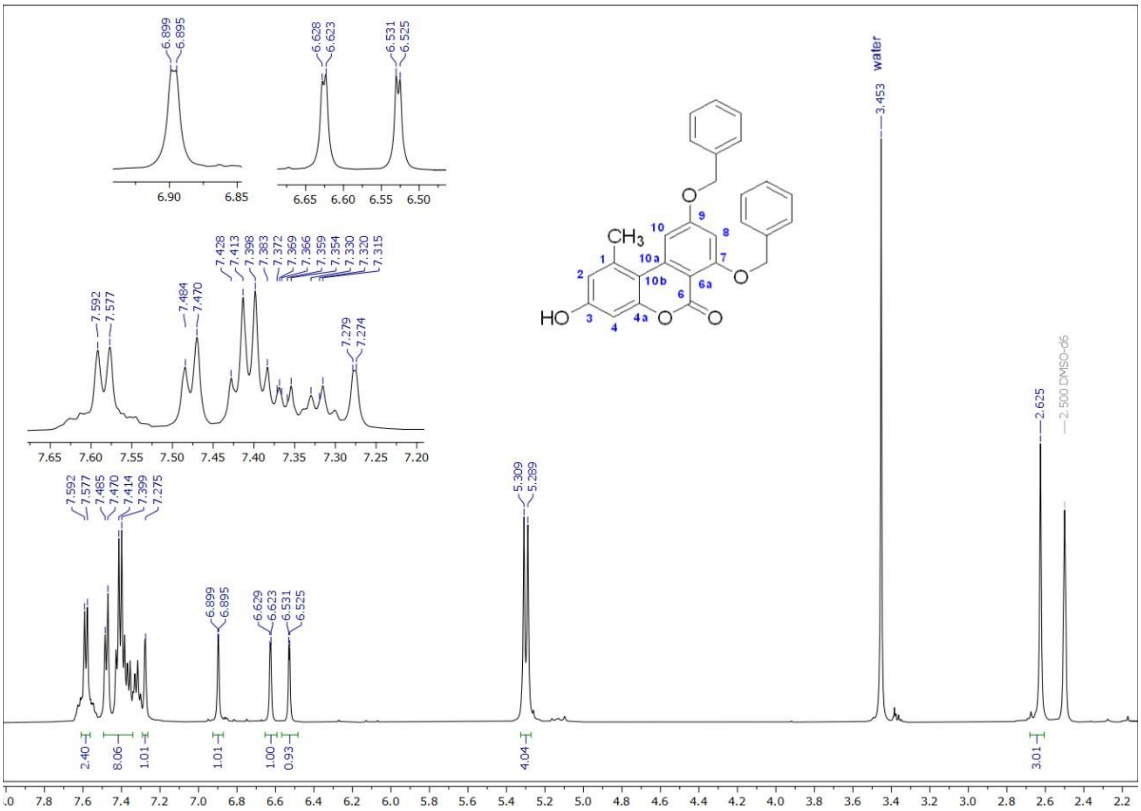

^{13}C NMR spectrum (126 MHz) of 7,9-dibenzyl ether of alternariol (**12**) in DMSO-d$_6$

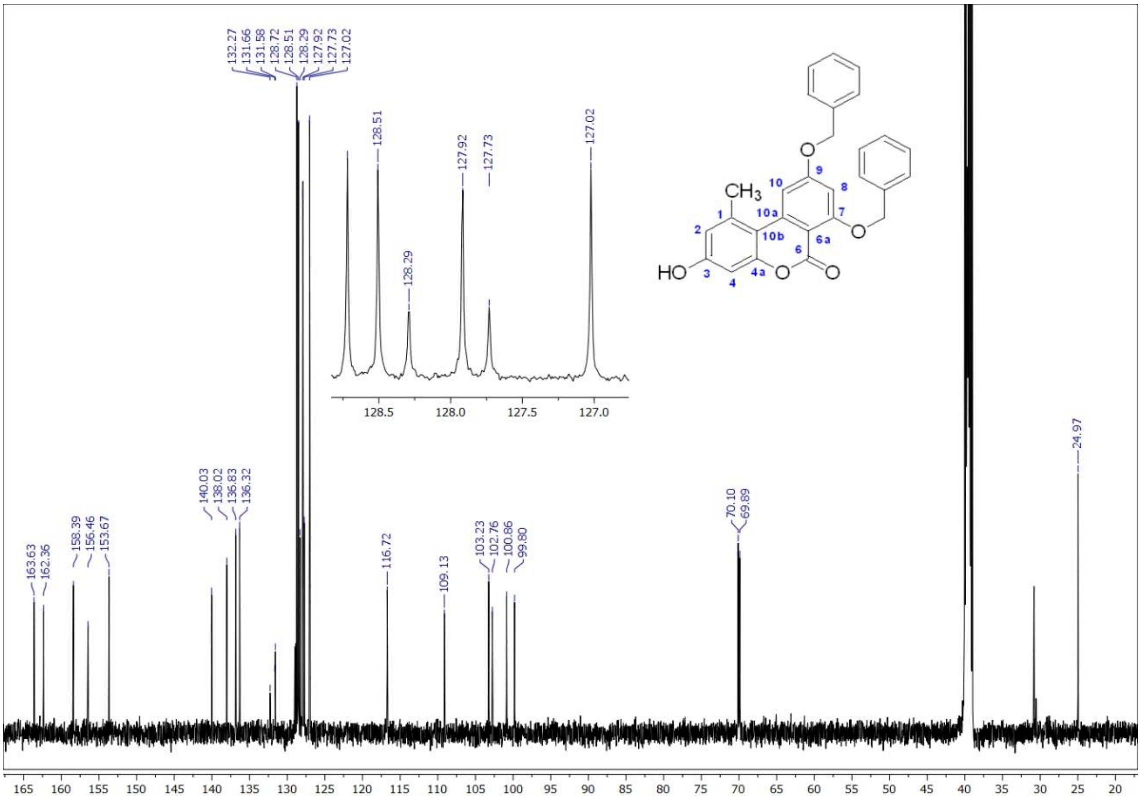

^{1}H NMR spectrum (500 MHz) of methyl ester **13** in $CDCl_3$

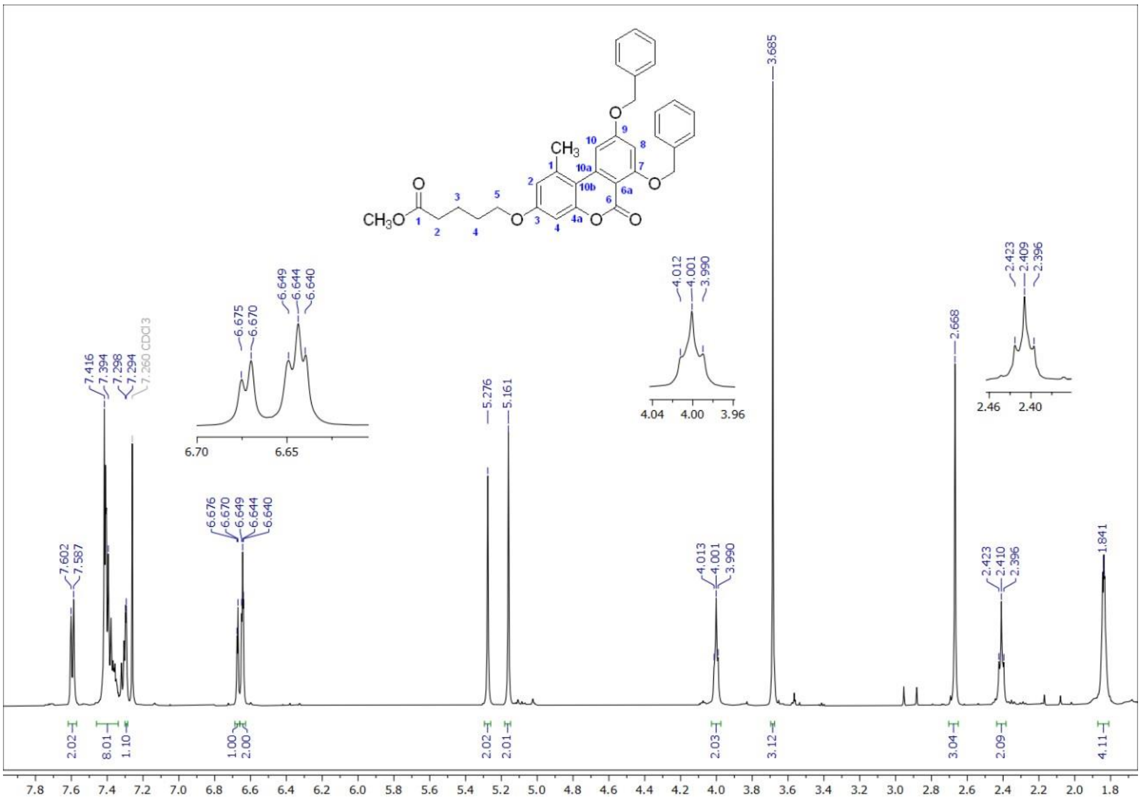

^{13}C NMR spectrum (126 MHz) of methyl ester **13** in $CDCl_3$

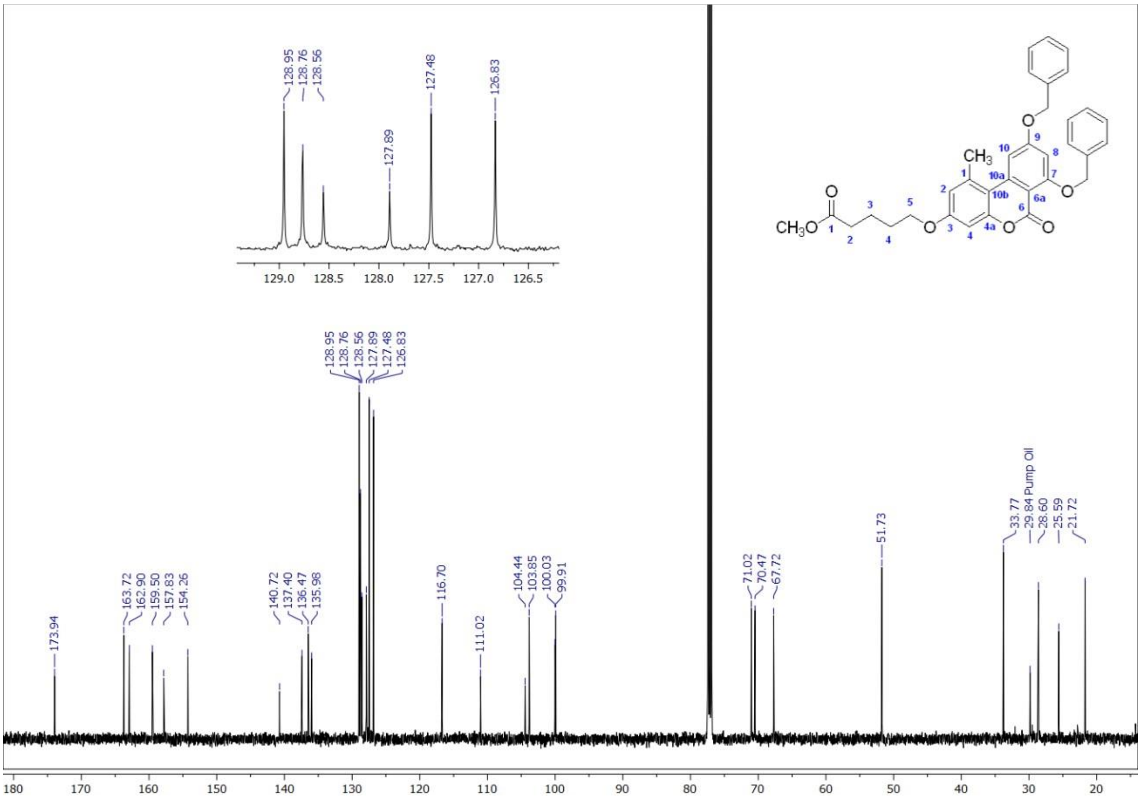

^{1}H NMR spectrum (500 MHz) of dibenzyl ether of hapten AL*b* (**14**) in THF-d$_8$

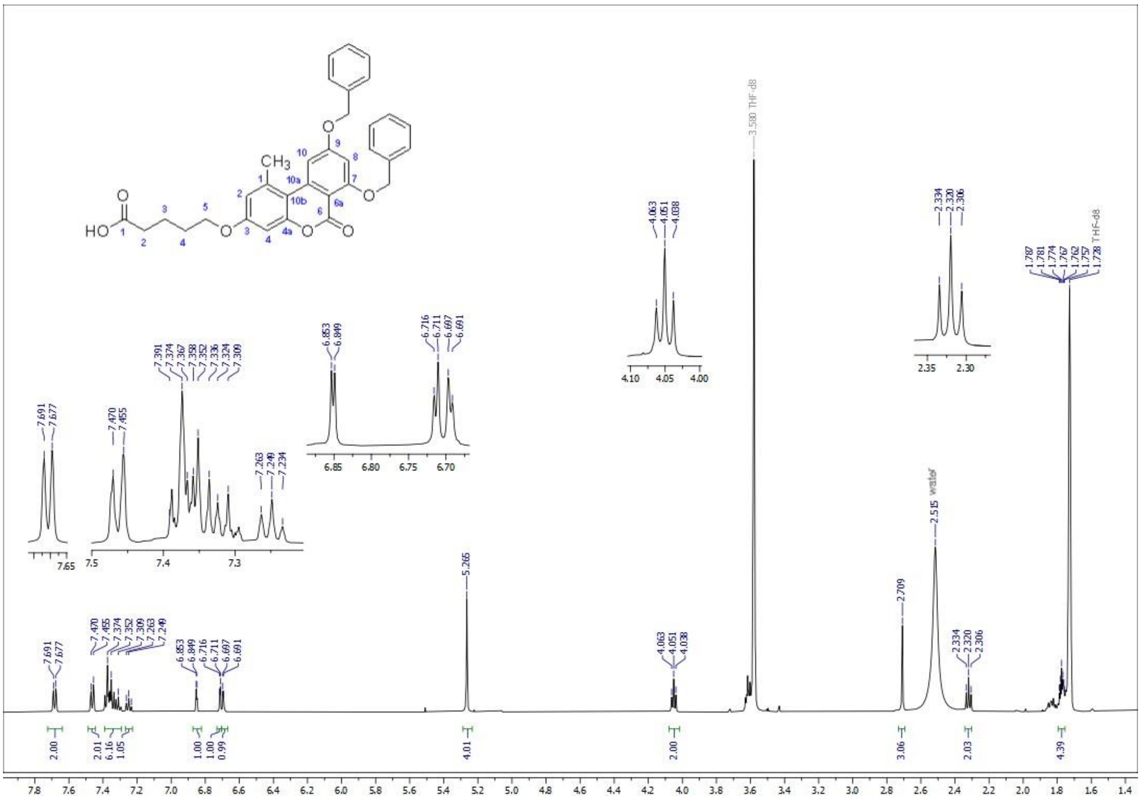

^{13}C NMR spectrum (126 MHz) of dibenzyl ether of hapten AL*b* (**14**) in THF-d$_8$

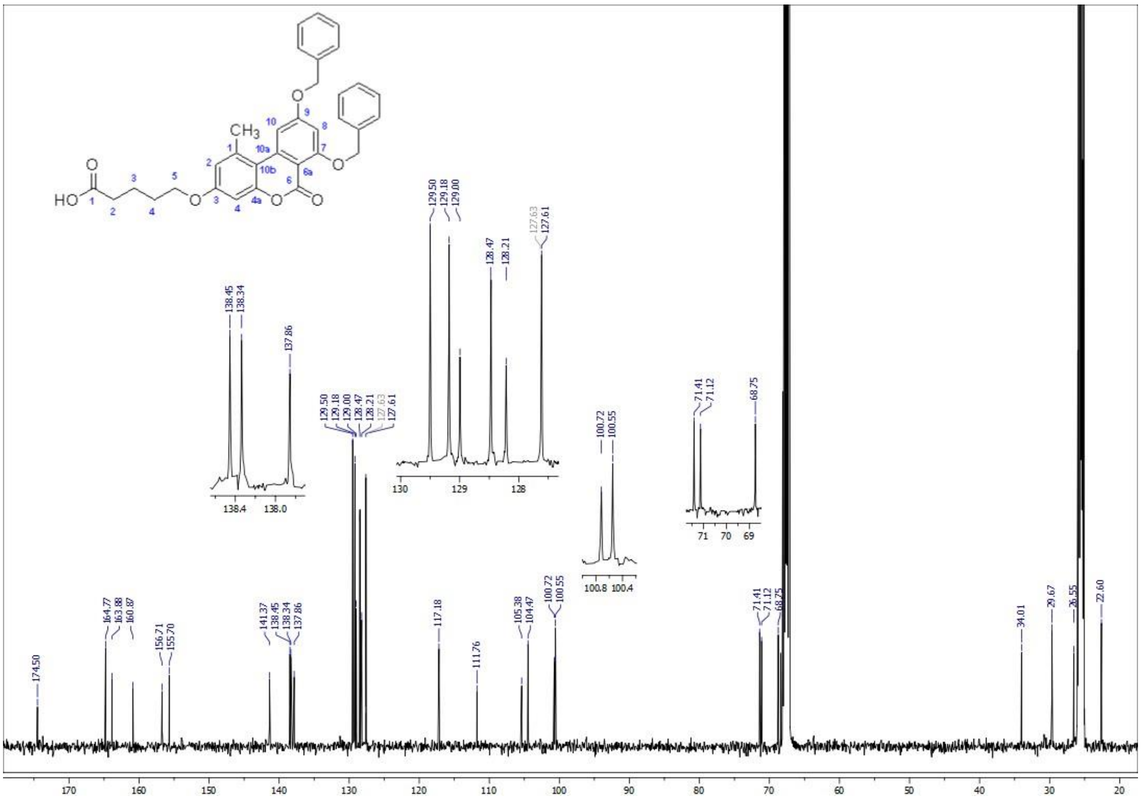

^1H NMR spectrum (300 MHz) of dibenzyl ether of AL*b*-NHS ester (**15**) in $CDCl_3$

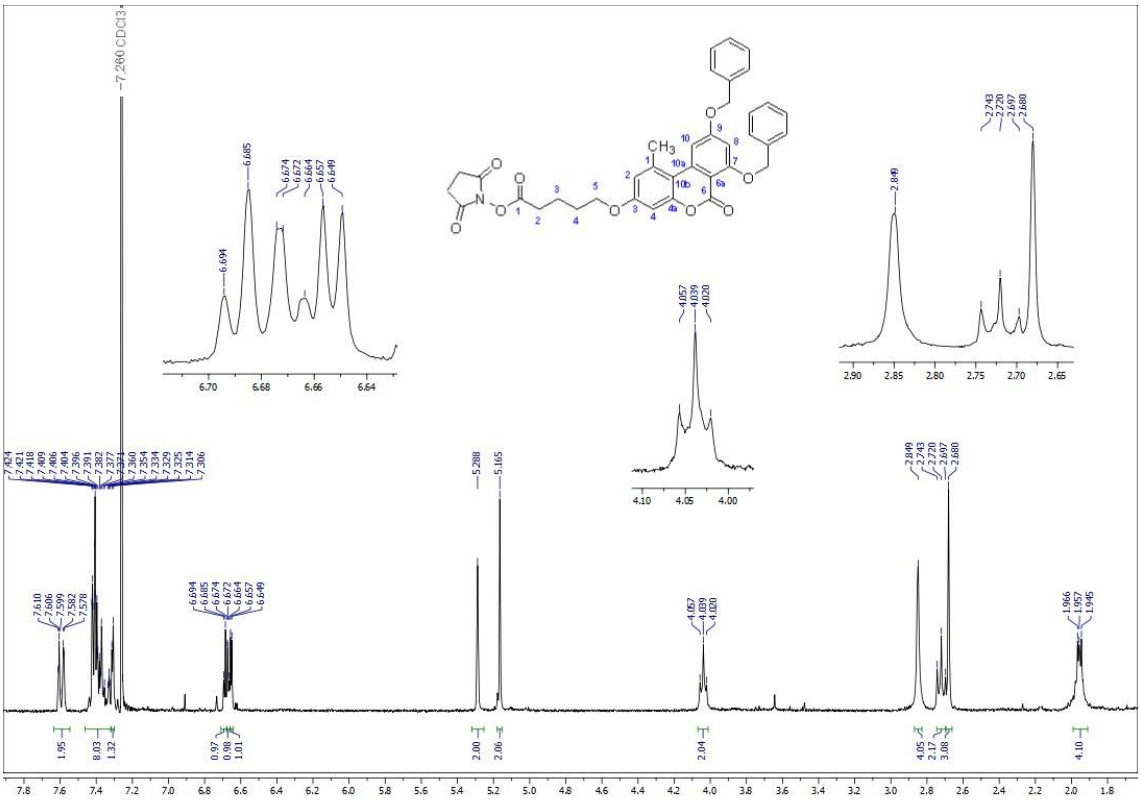

^1H NMR spectrum (500 MHz) of AL*b*-NHS ester in THF-*d*$_8$/DMSO-d$_6$

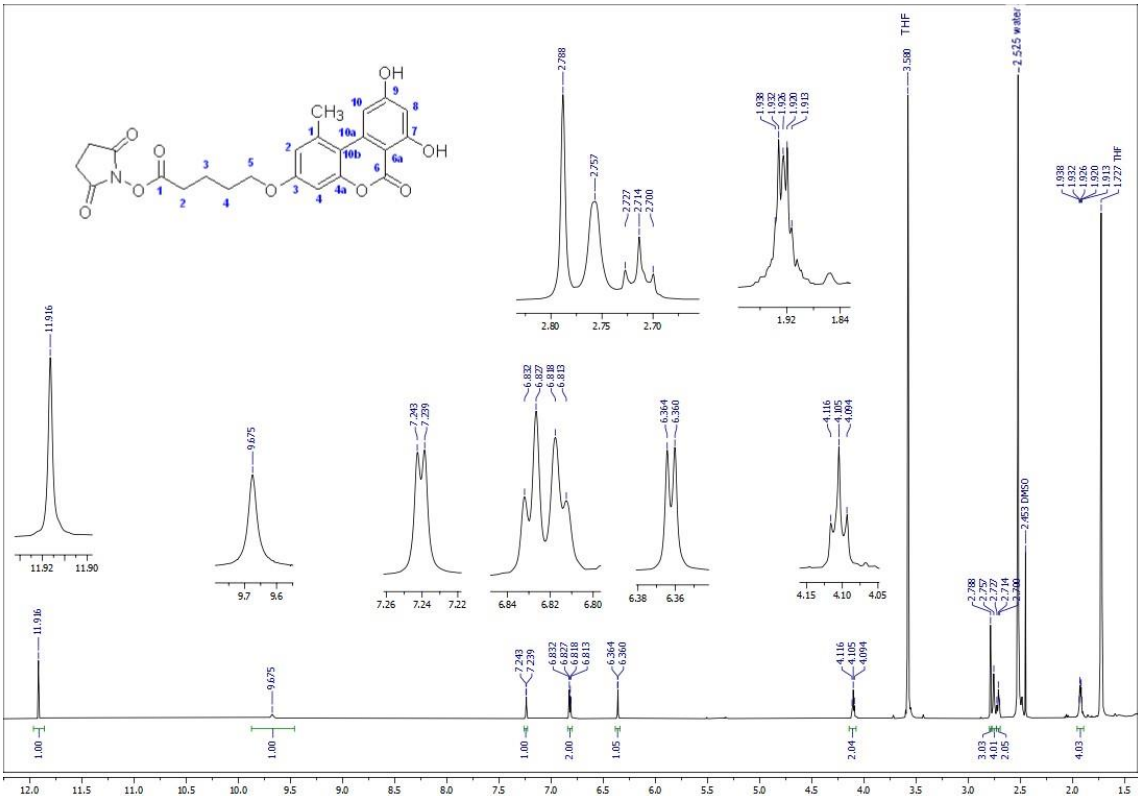

Supplement: Supplementary file 1 [file toxins-13-00883-s001.zip › toxins-1479242-supplementary.pdf]
